# Ultra-high throughput-based screening for the discovery of antiplatelet drugs affecting receptor dependent calcium signaling dynamics

**DOI:** 10.1038/s41598-024-56799-4

**Published:** 2024-03-14

**Authors:** Delia I. Fernández, Sara Troitiño, Vladimír Sobota, Bibian M. E. Tullemans, Jinmi Zou, Helma van den Hurk, Ángel García, Saman Honarnejad, Marijke J. E. Kuijpers, Johan W. M. Heemskerk

**Affiliations:** 1 The Department of Biochemistry, CARIM, Maastricht University, 6229 ER Maastricht, The Netherlands; 2 Platelet Proteomics Group, CiMUS, Universidade de Santiago de Compostela, 15782 Santiago de Compostela, Spain; 3 IHU-LIRYC, Electrophysiology and Heart Modeling Institute, Fondation Bordeaux Université, 33604 Bordeaux, France; 4 Institut de Mathématiques de Bordeaux, UMR5251, University of Bordeaux, 33 405 Talence, France; 5 Synapse Research Institute, Kon. Emmaplein 7, 6217 KD Maastricht, The Netherlands; 6 Pivot Park Screening Centre, 5349 AB Oss, The Netherlands; 7 Thrombosis Expertise Centre, Heart and Vascular Centre, Maastricht University Medical Centre+, 6229 HX Maastricht, The Netherlands

**Keywords:** Collagen, Cytosolic calcium, Glycoprotein VI, Prestwick library, Thrombin, Thrombosis, Calcium signalling, High-throughput screening, Platelets

## Abstract

Distinct platelet activation patterns are elicited by the tyrosine kinase-linked collagen receptor glycoprotein VI (GPVI) and the G-protein coupled protease-activated receptors (PAR1/4) for thrombin. This is reflected in the different platelet Ca^2+^ responses induced by the GPVI agonist collagen-related peptide (CRP) and the PAR1/4 agonist thrombin. Using a 96 well-plate assay with human Calcium-6-loaded platelets and a panel of 22 pharmacological inhibitors, we assessed the cytosolic Ca^2+^ signaling domains of these receptors and developed an automated Ca^2+^ curve algorithm. The algorithm was used to evaluate an ultra-high throughput (UHT) based screening of 16,635 chemically diverse small molecules with orally active physicochemical properties for effects on platelets stimulated with CRP or thrombin. Stringent agonist-specific selection criteria resulted in the identification of 151 drug-like molecules, of which three hit compounds were further characterized. The dibenzyl formamide derivative ANO61 selectively modulated thrombin-induced Ca^2+^ responses, whereas the aromatic sulfonyl imidazole AF299 and the phenothiazine ethopropazine affected CRP-induced responses. Platelet functional assays confirmed selectivity of these hits. Ethopropazine retained its inhibitory potential in the presence of plasma, and suppressed collagen-dependent thrombus buildup at arterial shear rate. In conclusion, targeting of platelet Ca^2+^ signaling dynamics in a screening campaign has the potential of identifying novel platelet-inhibiting molecules.

## Introduction

Antiplatelet medication forms the cornerstone in the secondary prevention of arterial cardiovascular diseases, but comes at the expense of non-negligible risks of bleeding^1,2^. Commonly used cyclooxygenase inhibitors (aspirin) and ADP receptor (P2Y_12_) inhibitors also have inadequate effects due to drug resistance or genetic polymorphisms^3–7^. The current pharmaceutical research & development interest focuses on developing antagonists for other target molecules, including the glycoprotein VI (GPVI) receptor for collagen and the protease-activated receptors 1 and 4 (PAR1, PAR4) for thrombin^2,8–11^.

Promising GPVI-directed inhibitors have been investigated in clinical trials, such as the recombinant GPVI-Fc construct Revacept^12,13^, and the humanized anti-GPVI antibody fragment Glenzocimab (ACT017)^14–16^. Regarding thrombin receptors, both an approved PAR1 antagonist vorapaxar^17^ and two PAR4 blockers (BMS-986120 and BMS-986141), profiled *ex vivo*^18,19^, show a promising antithrombotic potential. Accordingly, blocking platelet activation via the collagen and thrombin receptors is of pathophysiological importance for the secondary prevention of atherothrombosis, myocardial infarction and stroke^1,2^. However, a systematic search for compounds that can selectively interfere with GPVI- or PAR-induced platelet activation processes has not yet been carried out.

The ITAM-linked receptor GPVI and the G-protein coupled receptors PAR1/4 congregate in platelet responses, but differ in signaling by activating phospholipase C isoforms PLCγ2 and PLCβ, respectively^20,21^. Activation of both PLC forms leads to a rise in cytosolic [Ca^2+^]_i_, where GPVI/PLCγ2 stimulation results in a more sustained [Ca^2+^]_i_ signal than PAR/PLCβ stimulation^22^. Elevated [Ca^2+^]_i_ is known as a key second messenger in platelet activation, acting as an integration hub to other responses such as integrin activation, granule release, platelet aggregate formation and procoagulant activity^10^. Recently, we have developed a method to systematically compare agonist-induced [Ca^2+^]_i_ traces in 96-, 384- and 1,536-well plate formats^22^. This method allows for a large-scale ultra-high throughput (UHT) based screening of small molecule libraries, and holds the potential to identify novel platelet-inhibiting hit compounds with drug-like properties, *i.e.* with the physicochemical characteristics of an orally active drug^23^.

In the present study, we performed such a screening assay, based on the kinetics of platelet [Ca^2+^]_i_ rises to obtain small molecule compounds with selectivity towards the GPVI- or PAR-induced activation. For the screening, we used cross-linked collagen-related peptide (CRP) as a GPVI-selective agonist, and thrombin as a combined PAR1/4 agonist. Using a panel of established platelet inhibitors interfering with specific signaling domains, we developed a Ca^2+^ profiling algorithm to predict the modes of action per inhibitor. This algorithm was then applied to select hit molecules from a 16,635 small molecule screening performed in 1,536-well plates. Stringent agonist-specific selection criteria resulted in the identification of 151 drug-like molecules, of which three hit compounds were further characterized. We thus performed a proof-of-principle UHT-based screening campaign that targets the platelet Ca^2+^ signaling dynamics with the potential of identifying novel antiplatelet molecules.

## Results

### Dissecting GPVI- and PAR-induced signaling domains based on pharmacological inhibition of platelet Ca^2+^ responses

**Figure 1:**
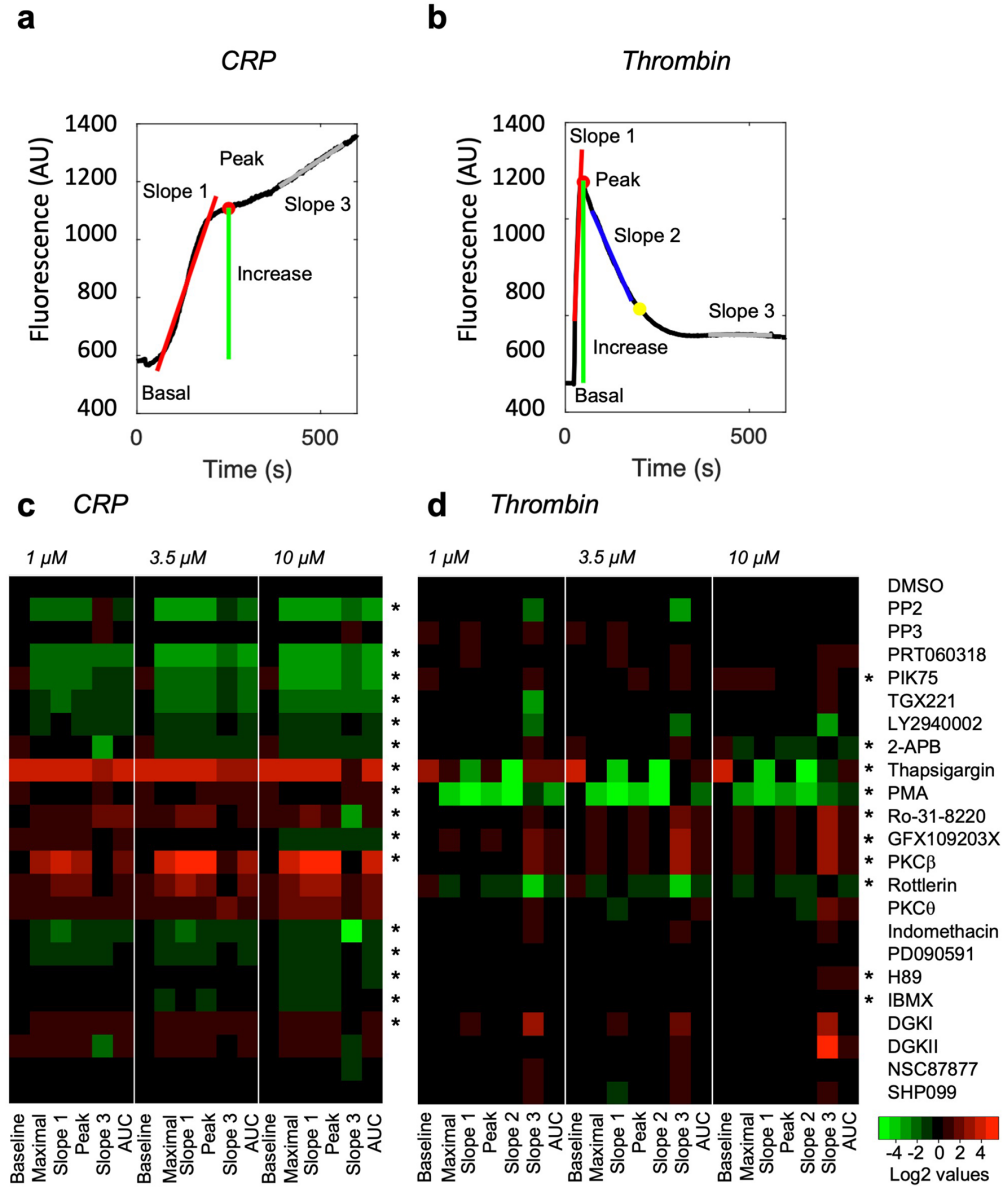
Cytosolic Ca^2+^ trace profiling and signaling domains of CRP- and thrombin-induced platelet activation. Calcium-6 loaded platelets (200 × 10^9^/L) were stimulated with CRP (10 µg/mL) or thrombin (4 nM) in 96-well plates. Platelets were preincubated with vehicle (DMSO) or one of the 22 indicated signaling inhibitors (Suppl. Table 1) at 1, 3.5 or 10 µM. (**a**,**b**) Representative fluorescence rises of Calcium-6 loaded platelets induced by CRP or thrombin, recorded by FlexStation 3. Color index: red line = interpolation of slope 1; red dot = peak level; green line = maximal cytosolic Ca^2+^ increase relative to the basal level; blue line = interpolation of slope 2; grey line = interpolation of slope 3. (c-d) Heatmap representation of log2 inhibitor effects on Ca^2+^ curve parameters (*P1*, baseline; *P2*, maximal Ca^2+^ increase; *P3*, slope 1; *P4*, peak Ca^2+^ rise; *P6*, slope 3, *P7*, area under the curve) for CRP (**c**) and thrombin (**d**). * Denotes inclusion in Reactome analysis, based on parameters with *P* < 0.05 (one-way ANOVA or Kruskal Wallis test). Data are from means of n = 3–4 platelet donors.

Platelet stimulation via the ITAM-linked receptor GPVI (via PLCγ2) and the G-protein coupled receptors PAR1/4 (via PLCβ) is known to give more prolonged or to shorter increases in [Ca^2+^]_i_, respectively^20,21^. The signaling pathways that contribute to these distinct response profiles are still partly unclear. To dissect this, we composed a panel of 22 pharmacological inhibitors with established mode of action towards specific signaling pathways in platelets (Table S1). The inhibitors were added to Calcium-6 loaded platelets at a dose range from 1, 3.5 to 10 μM, after which the cells were stimulated with a maximal concentration of CRP (10 µg/mL) or thrombin (4 nM). Fluorescence traces were collected in a 96-well plate setting, as described^22^. All Ca^2+^ response curves were then analyzed using a new automated algorithm, distinguishing seven curve parameters *P1-7* (Fig. 1a for 96-well plate, Fig. S1a for 1,536-well plate). The combination of parameters discriminated between the more prolonged CRP-induced Ca^2+^ response and the transiently peaking thrombin-induced response. A correlation matrix comparing [Ca^2+^]_i_ curves with all inhibitors indicated for CRP a high correlation between all parameters (except for baseline), in agreement with a strong and maintained signal (Fig. S1b). The correlation matrix for thrombin showed lower correlation for parameters of the curve slopes (*P3, P5-6)*.

Heatmapping of the effects of each inhibitor on the [Ca^2+^]_i_ curve parameters revealed patterns that were dependent on the type of drug and dose (Fig. 1b-c). For platelet stimulation with CRP, major and significant inhibitory effects were observed with the Syk kinase inhibitor PRT060318, the Src-family kinase inhibitor PP2, and the phosphoinositide 3-kinase (PI3K) inhibitors PIK75 and TGX221 (Fig. 1b). A small reduction, usually at the highest dose of 10 μM, was seen for the inositol trisphosphate receptor antagonist 2-ABP, the cyclooxygenase blocker indomethacin, the PI3K inhibitor LY294002, and the mitogen-activated protein kinase inhibitor PD98059. On the other hand, general inhibition of protein kinase C (PKC) isoforms (Ro-318220) or of only the isoform PKCθ resulted in a potentiated Ca^2+^ signal. The used PKCβ inhibitor also caused a stronger Ca^2+^ signal, likely because this compound also blocks other protein kinase C isoforms^24^. The Ca^2+^-ATPase inhibitor thapsigargin induced a [Ca^2+^]_i_ rise by itself, and enlarged the CRP-induced response.

Regarding the thrombin-induced Ca^2+^ response (Fig. 1c), most of the inhibitors had smaller effects than seen with CRP. Consistent inhibition was present upon InsP_3_ receptor inhibition, PKC stimulation with phorbol ester PMA, or with the PKCδ inhibitor rottlerin. Other PKC-inhibiting compounds (Ro-318220, GF109203X, PKCβ inhibitor) increased one or more parameters of the thrombin-induced Ca^2+^ signal. Inhibitors targeting phosphodiesterases, tyrosine phosphatases SHP1 or SHP2, or diacylglycerol kinase did not or hardly cause alterations in the Ca^2+^ responses.

To translate these results to established signaling pathways, we performed a Reactome pathway analysis with as input the proteins targeted by those inhibitors showing a significant inhibiting or potentiating effects of the platelet Ca^2+^ signals. For CRP, the analysis resulted in 49 over-represented pathways surpassing the set thresholds (n ≥ 4 entities, false discovery rate FDR < 1%) (Table S2). For thrombin, this resulted in 46 over-represented pathways based on the same thresholds. Reactome results per receptor type showed multiple overrepresented pathways, such as hemostasis; signal transduction; signaling by GPCR and downstream; activation, signaling and aggregation of platelets; effects of PIP_2_ hydrolysis and InsP_3_ signaling; and signaling by receptor tyrosine kinases. Overrepresented pathways for only CRP were PECAM1 interactions, interleukin signaling activation of B cell receptors, and PI3K/AKT signaling. Present in only the thrombin list was, *e.g.,* signaling by Rho and other GTPases. Taken together, this analysis pointed to a partly different contribution of platelet signaling pathways in the integrative Ca^2+^ responses induced by GPVI or PAR1/4 stimulation.

### Ultra-high throughput based small molecule screening for potential hit compound identification

**Figure 2:**
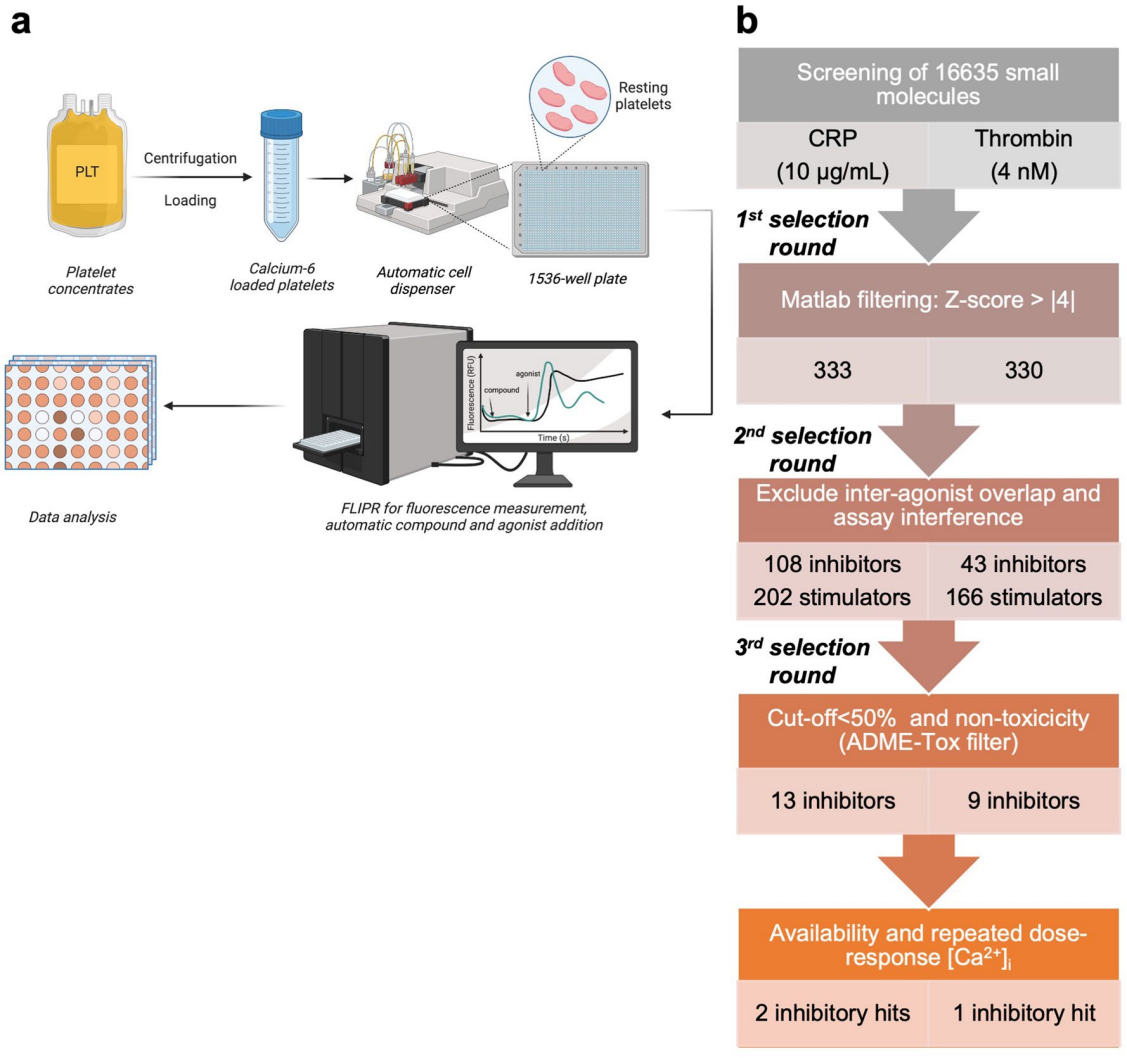
Screening of small molecules for effect on platelet Ca^2+^ rises and hit selection workflow. (**a**) Schematic representation of screening workflow, and (**b**), hit selection workflow via filtering for Z-scores, agonist-specific effect, non-toxicity, and repeated dose–response [Ca^2+^]_i_ measurements.

Recently, we described a miniaturized, UHT assay to measure [Ca^2+^]_i_ rises in platelets induced by CRP or thrombin in 1536-well plate format^22^. This miniaturized assay, using 4 μL Calcium-6-loaded platelets, was used for a large screening test to find novel compounds with selectivity towards the platelet ITAM-linked receptor GPVI (CRP, 10 μg/mL) or the GPCR PAR1/4 (thrombin, 4 nM) in the presence of 1 mM CaCl_2_. For either agonist, we tested a total of 16,635 small organic molecules in duplicate well plates (Datafile S1). The molecules were composed of the Prestwick chemical library of 1,280 off-patent, FDA-approved compounds^25,26^; and a diversity-based library consisting of 15,355 small molecule compounds (SMC). The SMC set contained compounds with all drug-like properties in terms of solubility, hydrogen bonds, topological polar surface, and non-hydrogen/non-carbon atoms (Fig. S2a). All compounds were tested in 1,536-well plates at a final concentration of 10 µM (Fig. 2a).

The obtained duplicate (~ 90%) fluorescent [Ca^2+^]_i_ traces per agonist and compound were automatically analyzed using the seven-parameter curve profiling algorithm (Datafile S1). In order to check for consistency, correlation analysis was performed of the complete dataset for the *P1-7* parameter values per agonist (Fig. S1c). Matrix heatmapping for CRP responses showed positive correlations between all parameters, except for a negative correlation with the baseline (*P1*, due to baseline compound effects) and a lower correlation for the late slope (low *P6*). Heatmapping for thrombin also showed high positive correlations for the non-baseline parameters *P2-7*.

Using a custom-made algorithm written in Matlab, we compared the multiparameter curve characteristics *P1-7* to select for hit compounds (Fig. 2b), first by filtering per agonist for consistent duplicates and then for Z-scores >|4| (Fig. S3). This stringent filtering for reproducibility and effect size resulted in the selection of so-called ‘active’ compounds. The number of active compounds with CRP (n = 333) or thrombin (n = 330) was subsequently confined to compounds that affected the Ca^2+^ responses with only one agonist. This resulted in 310 compounds for CRP and 209 compounds for thrombin. After removing compounds that increased rather than decreased the [Ca^2+^]_i_ rises, we obtained 108 and 43 inhibitors (Fig. S2b-c) of CRP- and thrombin-induced Ca^2+^ responses, respectively (Fig. 2b). The curves were then visually inspected for abnormalities, *i.e.* for incorrect agonist injection or compound interference with the fluorescent signal. For CRP (thrombin), this led to rejecting 17 (8) compounds due to incorrect agonist injection and to 55 (22) fluorescence-interfering compounds. Furthermore, we filtered for in silico estimation of compounds with toxicity or unfavorable pharmacokinetics according to ADMET criteria (absorption, distribution, metabolism, excretion and toxicity)^27^. For the CRP (thrombin) set, 23 (4) compounds showed ADME-Tox characteristics, and these were hence rejected.

As a result, we obtained 13 inhibitors for CRP and 9 inhibitors for thrombin, including 5 compounds from the Prestwick set (Fig. S4), which after decoding appeared to be commercially available. Three of the Prestwick compounds were not further investigated due to known antiplatelet effects^28^, *i.e.* the cyclooxygenase inhibitors ketoprofen and valdecoxib (effect with CRP), and a derivative of the P2Y_12_-inhibiting clopidogrel (effect with thrombin). Other Prestwick compounds with unfavorable side effects^28^ were the anthelmintics nitazoxanide (CRP) and triclabendazole (thrombin), and the anesthetics bupivacaine, hydroxyzine and oxethazaine (thrombin). As hits for further studies, we selected the phenothiazine derivatives ethopropazine (PRES000840, used for Parkinson treatment) and ethaverine (PRES000830) as inhibitors of CRP-induced platelet activation. In addition, from the SMC library, 8 and 4 compounds were purchased targeting the CRP and thrombin responses, respectively (Fig. S5).

### Further validation of active and hit compounds

Testing of the 10 compounds for CRP and the 4 compounds for thrombin (standard dose of 10 µM) on platelet Ca^2+^ responses in a 96-well plate assay confirmed > 25% inhibitory effects for 5 and 2 compounds, respectively (Fig. S5a,b). As a next step, we determined the dose–response relationship by re-testing the remaining 7 compounds at the higher dose of 30 μM (Fig. S5c–d). This finally resulted in three hit compounds with requested inhibitory properties, namely ANO61 (SPCA054640_01, thrombin), AF299 (SPCA056289_01, CRP) and ethopropazine (profenamine, CAS 522–00-9, CRP), all with reactive N electrons and an aromatic chemical structure.

**Figure 3:**
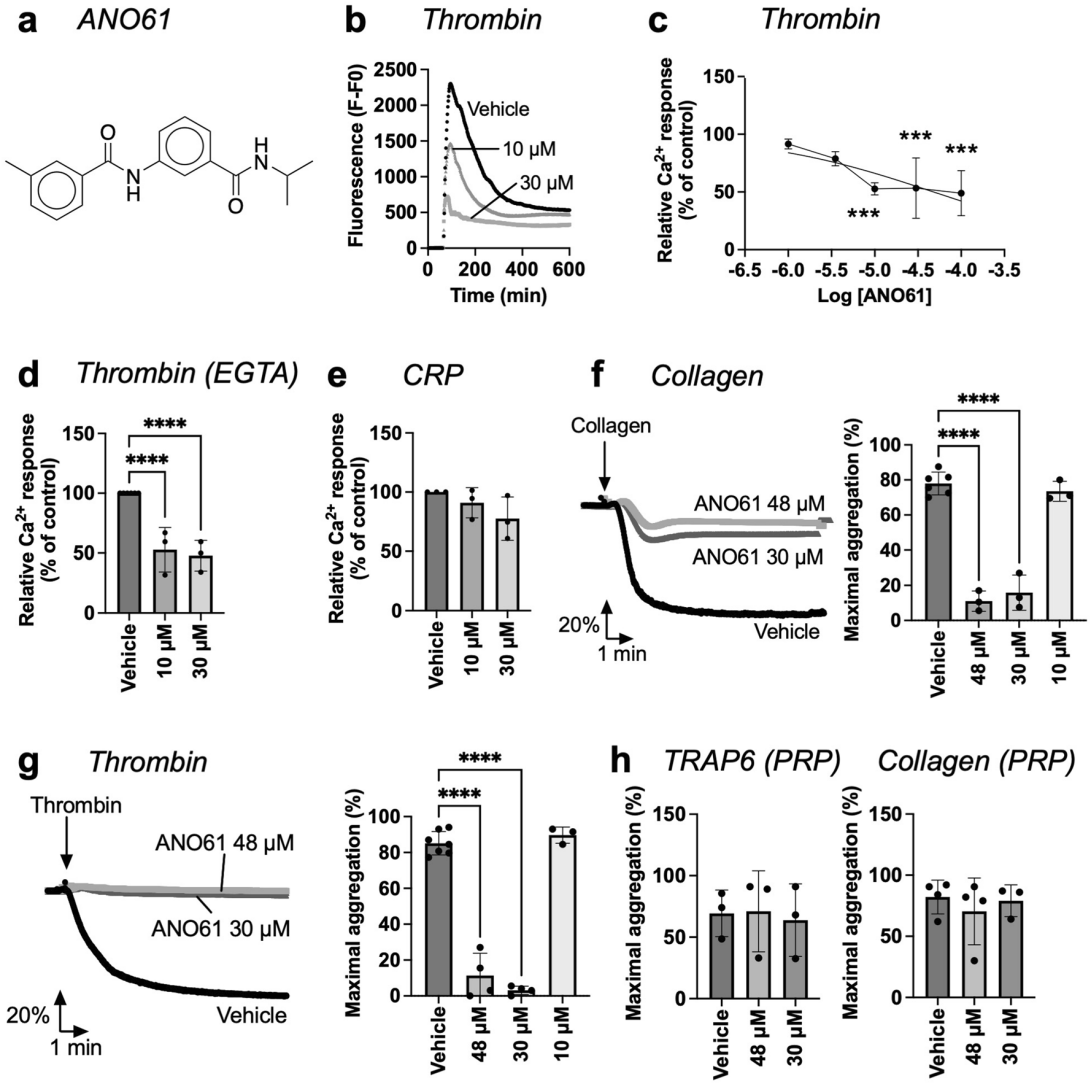
ANO61 reduced thrombin-induced platelet Ca^2+^ rises. (**a**) Chemical structure of ANO61. (**b**–**e**) Calcium-6 loaded platelets (200 μL, 200 × 10^9^/L) in 96-well plates were stimulated with thrombin (4 nM) or CRP (10 µg/mL) after 10 min preincubation with vehicle medium or indicated concentrations of ANO61. Fluorescence changes from wells were recorded using a FlexStation 3 machine. (**b**) Representative thrombin-induced [Ca^2+^]_i_ response curves in the presence of 1 mM CaCl_2_. (**c**) Dose–response effects on the maximal Ca^2+^ responses (parameter P*2*), normalized to the control condition. (**d**) Effect of ANO61 on thrombin-induced Ca^2+^ responses in the presence of 0.1 mM EGTA, blocking extracellular Ca^2+^ entry. (**e**) Selectivity was studied using CRP-induced Ca^2+^ responses after ANO61 incubation. (**f**,**g**) Effect of ANO61 on platelet aggregation in washed platelets (250 × 10^9^/L) in response to collagen (1 µg/ml, *f*) or thrombin (1 nM, *g*). Pretreatment was for 10 min with vehicle medium or ANO61 (10, 30, 48 µM). Shown are representative light transmission traces and histograms of maximal aggregation. (**h**) PRP (250 × 10^9^/L) preincubated with ANO61 (30–48 µM) was stimulated with 15 µM TRAP6 or 1 µg/mL collagen, and platelet aggregation was measured. Shown are maximal aggregation values. Data are presented as mean ± SD (n = 3–4 donors); ****P < 0.0001, one-way ANOVA with Tukey post-hoc test.

The dibenzyl formamide derivative ANO61 showed dose-dependent inhibitory effects on [Ca^2+^]_i_ curves induced by 4 nM thrombin with an IC_50_ of 47.7 µM (Fig. 3a–c). This effect was enlarged at a lower thrombin dose of 2 nM (Fig. S6a) and persisted in the presence of extracellular EGTA, thus indicating suppression independently of extracellular Ca^2+^ entry (Fig. 3d). As expected, ANO601 did not show inhibition with CRP as agonist (Fig. 3e). Furthermore, ANO61 appeared to be non-toxic for platelets at 100 μM (Fig. S6d). When testing ANO61 (30–49 μM) on the functions of washed platelets, we observed a significant reduction of the collagen-induced (Fig. 3f) and thrombin-induced (Fig. 3g) platelet aggregation. We also monitored platelet aggregation in the presence of plasma—*i.e.* a condition which often requires higher concentrations for effective response suppression^29,30^. With plasma present, ANO61 at the highest dose of 48 μM did no longer affect the aggregation responses by collagen (GPVI agonist) or TRAP6 (PAR1 agonist) (Fig. 3h).

**Figure 4:**
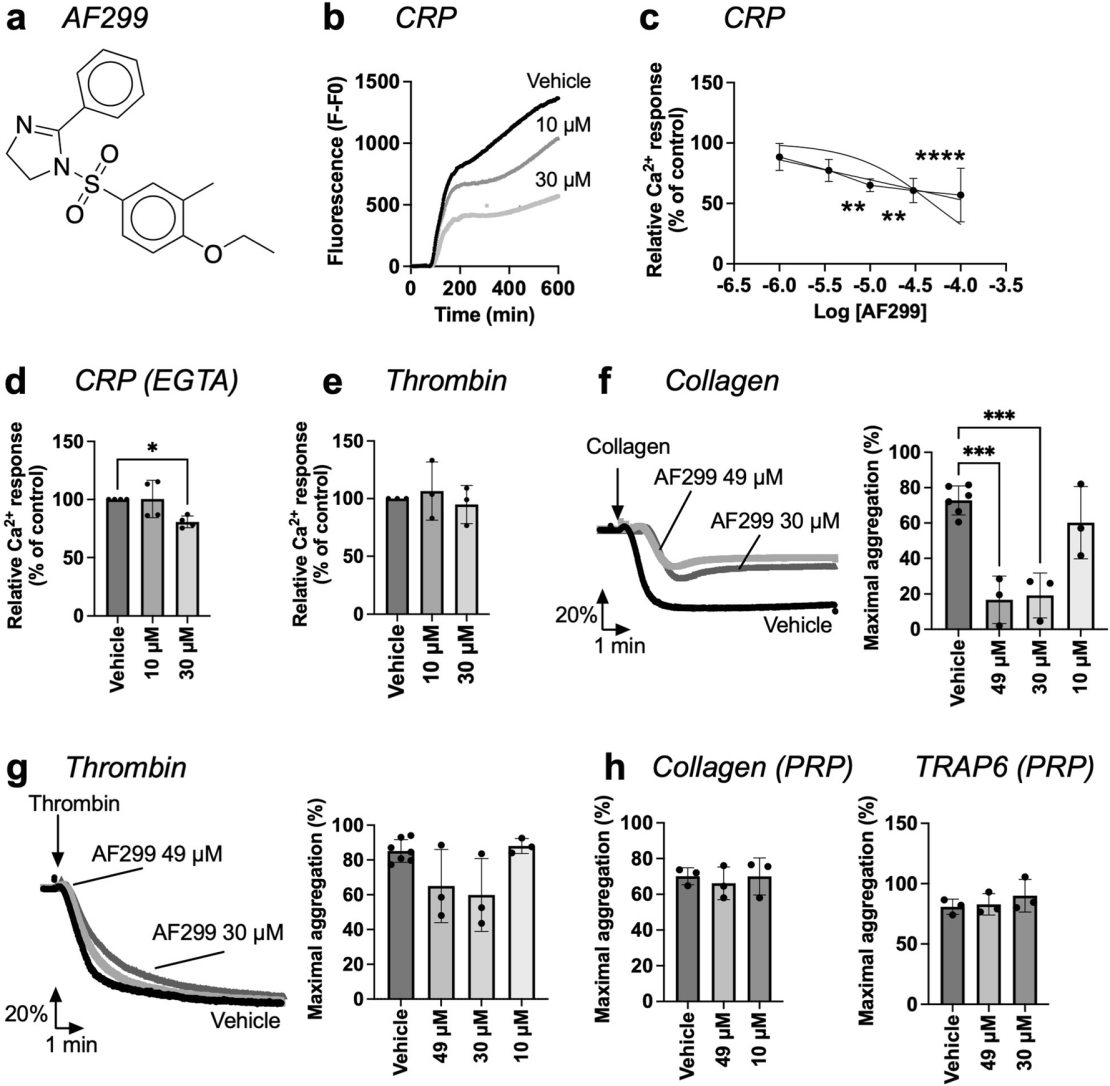
AF299 reduced CRP-induced platelet Ca^2+^ rises and collagen-induced platelet aggregation. (**a**) Chemical structure of AF299. (**b**–**e**) Calcium-6 loaded platelets in 96-well plates were stimulated with CRP (10 µg/mL) or thrombin (4 nM), as for Fig. 3. Preincubation was with vehicle medium or indicated concentrations of AF299. Fluorescence changes from wells were recorded using a FlexStation 3 machine. (**b**) Representative CRP-induced [Ca^2+^]_i_ response curves in the presence of 1 mM CaCl_2_. (**c**) Dose–response effects on the maximal Ca^2+^ responses (parameter P2), normalized to the control condition (DMSO). (**d**) Effect of AF299 on CRP-induced Ca^2+^ responses in the presence of 0.1 mM EGTA, blocking extracellular Ca^2+^ entry. *(e)* Selectivity of AF299 was studied for not affecting the Ca^2+^ responses induced by the other agonist (thrombin). (**f**,**g**) Effect of AF299 on platelet aggregation in washed platelets in response to collagen (1 µg/mL, f) or thrombin (1 nM, g). Pretreatment was for 10 min with vehicle medium or AF299 (10, 30, 49 µM). Shown are representative light transmission traces and histograms of maximal aggregation. (**h**) PRP preincubated with AF299 (30–49 µM) was stimulated with 1 µg/mL collagen or 15 µM TRAP6, and platelet aggregation was measured. Data are presented as mean ± SD (n = 3–4 donors); *P < 0.05, **P < 0.01, ***P < 0.001 ****P < 0.0001, one-way ANOVA with Tukey post-hoc test.

The aromatic sulfonyl imidazole AF299 suppressed the platelet Ca^2+^ response induced by 10 μg/mL CRP at an IC_50_ of 48.6 μM (Fig. 4a–c). AF299 was similarly effective at a lower dose of 5 μg/mL CRP (Fig. S6b), without causing platelet toxicity (Fig. S6e). The AF299-induced inhibition of the Ca^2+^ signal with CRP remained in the presence of EGTA, most selective at the lower concentration (Fig. 4d), whereas no effect was seen upon thrombin stimulation (Fig. 4e). Platelet aggregation measurement at concentrations ≥ 30 µM showed a significant reduction upon stimulation with collagen, but not with thrombin (Fig. 4f,g). However, in platelet-rich plasma, pre-incubation of 30–49 μM AF299 no longer inhibited aggregation induced by collagen or TRAP6 (Fig. 4h).

**Figure 5:**
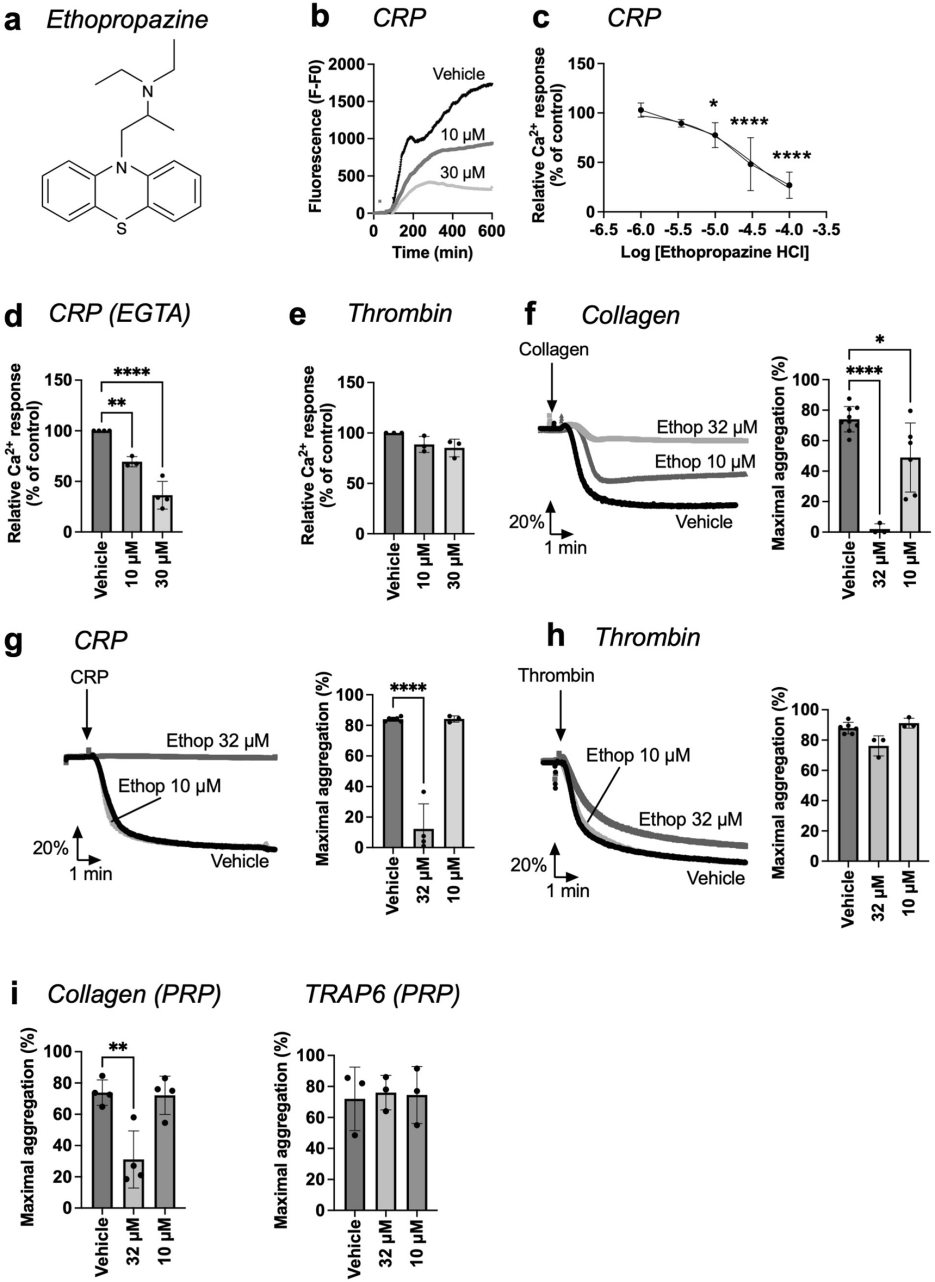
Suppressive effect of ethopropazine on platelet functions. (**a**) Chemical structure of ethopropazine. (**b**–**e**) Calcium-6 loaded platelets in 96-well plates were stimulated with or CRP (10 µg/mL) or thrombin (4 nM), as for Fig. 3. Preincubation with vehicle or indicated concentrations (1, 3.5, 10, 30, 100 µM) of ethopropazine (Ethop). Fluorescence changes from wells were recorded using a FlexStation 3 machine. (**b**) Representative CRP-induced [Ca^2+^]_i_ response curves in the presence of 1 mM CaCl_2_. (**c**) Dose–response effects on the maximal Ca^2+^ responses (parameter P2), normalized to the control condition. (**d**) Effect of ethopropazine on CRP-induced Ca^2+^ responses in the presence of 0.1 mM EGTA, blocking extracellular Ca^2+^ entry. (**e**) Selectivity was studied using thrombin-induced Ca^2+^ responses after ethopropazine incubation. (**f**–**h**) Effect of ethopropazine on platelet aggregation in washed platelets in response to collagen (1 µg/mL, f), CRP (10 µg/mL, g), or thrombin (4 nM, h). Pretreatment was for 10 min with vehicle medium or ethopropazine (10 and 32 µM). Representative light transmission traces and histograms of maximal aggregation are shown. (**i**) Platelet aggregation of PRP preincubated with ethopropazine (10–32 µM) and stimulated with 1 µg/mL collagen, or 15 µM TRAP6. Shown are maximal changes in light transmission. Data are presented as mean ± SD (n = 3–5 donors); *P < 0.05, **P < 0.01, ****P < 0.0001, one-way ANOVA with Tukey pot-hoc test.

The phenothiazine ethopropazine, re-assayed in 96-well plates, showed a dose-dependent inhibition of the maximal Ca^2+^ signal induced by 10 μg/mL CRP with an IC_50_ of 31.7 μM (Fig. 5a–c). The inhibition persisted at the lower dose of 5 μg/mL CRP (Fig. S6c) and in the presence of EGTA (Fig. 5d). No toxicity on platelets was observed (Fig. S6f). As required, ethopropazine did not affect the Ca^2+^ signal of thrombin-stimulated platelets (Fig. 5e). Importantly, at concentrations of 10–32 μM ethopropazine significantly and dose-dependently suppressed the collagen- and CRP-induced platelet aggregation responses (Fig. 5f,g), but it failed to affect the thrombin-induced aggregation (Fig. 5h). When testing in platelet-rich plasma, 32 μM ethopropazine again markedly suppressed the aggregation with only collagen, but not with TRAP6 (Fig. 5i). Together, these observations are consistent with a selective, physiologically relevant suppressive effect of ethopropazine on GPVI-induced platelet activation processes.

### Validating effects of ethopropazine on platelet responses

**Figure 6:**
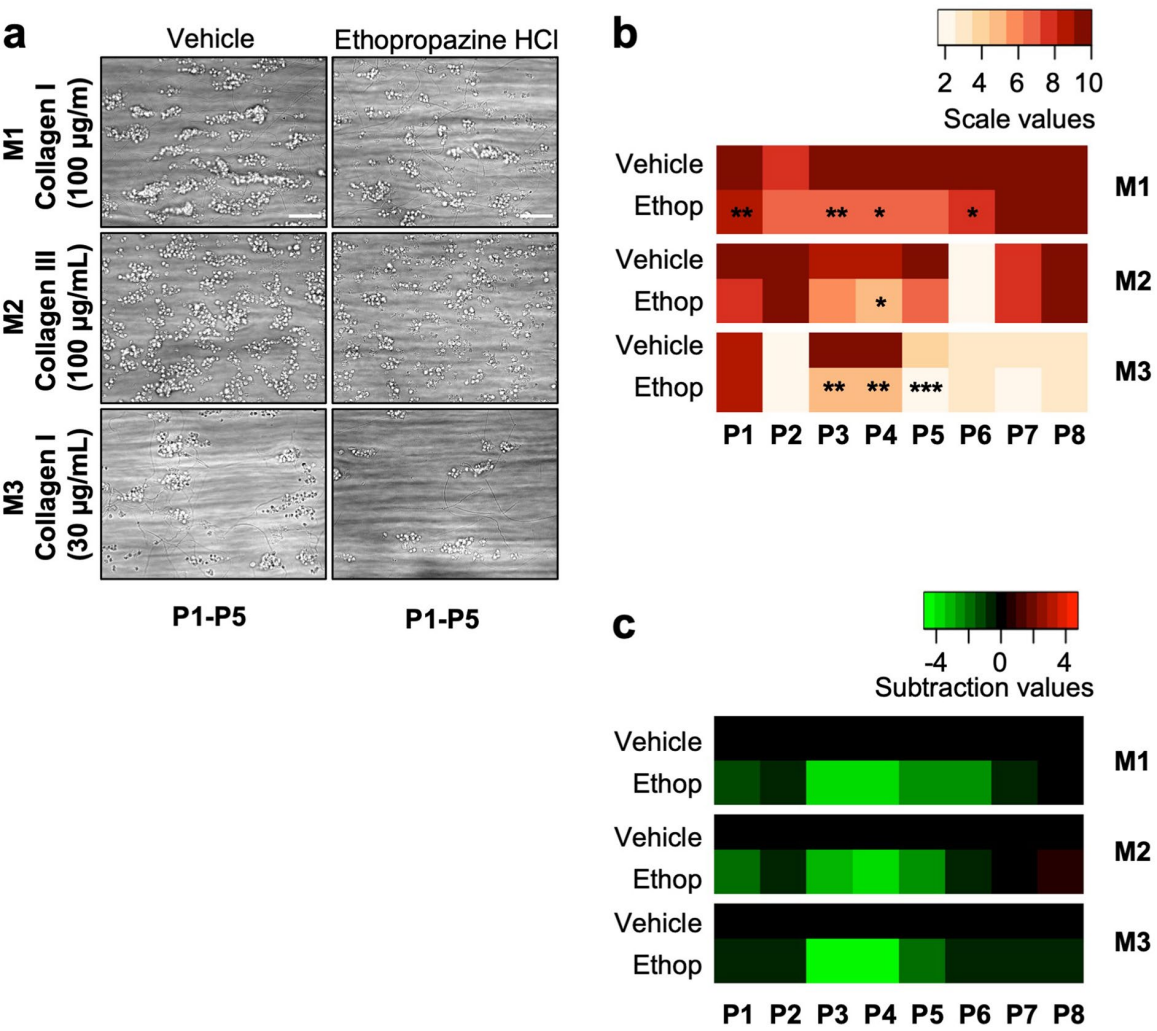
Ethopropazine inhibits collagen-induced whole-blood thrombus formation under flow. (**a**–**c**) Effect of ethopropazine on platelet thrombus formation under flow. Blood samples were preincubated with vehicle or ethopropazine (32 µM). After recalcification, the samples were perfused for 3.5 min at a wall-shear rate of 1000 s^−1^ over microspots of collagen I (100 µg/mL, M1), collagen III (100 µg/mL, M2), and collagen I (30 µg/mL, M3). (**a**) Representative brightfield microscopic images from indicated microspot surfaces, used for analysis of thrombus parameters *P1-P5*. Scale bars = 20 µm. (**b**) Heatmap of scaled parameters per microspot surface, indicating ethopropazine effects with statistics. Mean values of quantified 8 parameters were univariate scaled (0–10) across M1-M3. Parameters are defined as: *P1*, thrombus morphological score *P2*, platelet surface area coverage (SAC%); *P3*, thrombus contraction score; *P4*, thrombus multilayer score, and *P5*, platelet multilayer coverage (SAC%); *P6*, phosphatidylserine exposure (SAC%); *P7*, P-selectin expression (SAC%); and *P8*, integrin αIIbβ3 activation (SAC%). (**c**) Subtraction heatmap representing scaled ethopropazine effects. Color code represents decrease (green) or increase (red) in comparison to control blood flow runs. Data are means (n = 3–5 donors). *P < 0.05, **P < 0.01, ***P < 0.001, vs. vehicle, paired Student t-test, see Datafile S1. For individual parameter values, see Figure S7.

The results so far prompted us to assess the effects of ethopropazine on platelet aggregation under conditions of shear-dependent thrombus formation in whole blood. For this purpose, we used a previously validated microfluidic system with coated microspots of vascular-type collagens I and III, over which a recalcified blood sample was perfused at defined arterial wall-shear rate of 1000 s^−1^ ^31^. We found that the addition of 32 μM ethopropazine resulted in a substantial reduction in the formation of platelet thrombi on both collagen surfaces (Fig. 6a). Univariate scaling of the eight quantified thrombus parameters indicated that ethopropazine significantly reduced parameters of thrombus size, contraction and structure, specifically on collagen I (Fig. 6b,c, full data in Fig. S7). This result extended and agreed with the observed effect on platelet aggregation measurements.

To explore the mechanism of action of ethopropazine, we compared its effect on CRP-induced [Ca^2+^]_i_ curve parameters with the earlier observed effects of 22 signaling pathway inhibitors (see Fig. 1). Using K-means clustering and principal component analysis, we found several similarities in Ca^2+^ response modulation (Fig. S8a). The obtained matrix of Euclidean distances using reference compounds and ethopropazine indicated similarity with the inhibition of PI3K, inositol trisphosphate receptors, MAP kinases, protein kinase A and phosphodiesterases (Fig. S8b).

To take this further, we assessed the Ca^2+^ response effects of ethopropazine in combination with indomethacin and apyrase (blocking secondary mediator pathways), which revealed additional inhibition by ethopropazine (Fig. S9a). Co-treatment of the platelets with modulators of cAMP (*i.e.* phosphodiesterase inhibitor IBMX and adenylyl cyclase inhibitor SQ22536) also retained the effect of ethopropazine (Fig. S9b). Furthermore, in combination with inhibitors of PI3Kα (PIK75) and β (TGX221), ethopropazine was no longer effective (Fig. S8c). In order to check for signaling events immediately downstream of GPVI, we examined the CRP-induced protein tyrosine phosphorylation pattern with 4G10 antibody, and also checked for the phosphorylation of PLCγ2 at Y^259^ and Syk at Y^525/526^ ^32,33^. Ethopropazine treatment (10–32 μM) did not appear to influence these CRP-induced phosphorylation events (Fig. S8d), thus excluding a direct effect on the Src-Syk-PLCγ2 pathway. Taken together, these data suggest that ethopropazine likely interferes with the PI3K route of the GPVI-induced signaling downstream of PLCγ2. This is in agreement with a demonstrated role of PI3Kα, β and δ isoforms in platelet Ca^2+^ responses and thrombus formation, such as reported before^29,34^.

## Discussion

In this report, we used a novel algorithm for profiling the [Ca^2+^]_i_ curves of Calcium-6-loaded platelets stimulated by the GPVI agonist CRP or with PAR1/4 agonist thrombin to aid in the analysis of systematic screening for novel platelet-inhibiting compounds. After UHT-based screening of 16,635 small molecules (with drug-like physicochemical characteristics) for effects on agonist-induced [Ca^2+^]_i_ curve parameters, we obtained 151 agonist-specific drug-like molecules after applying stringent selection criteria. Further compound prioritization and confirmation measurements ultimately resulted in three hits, *i.e.* two compounds affecting Ca^2+^ rises as well as platelet aggregation induced by CRP (AF299 and ethopropazine), and one compound interfering with platelet activation by thrombin (ANO61). Given their half-maximal effective concentrations of > 10 μM, it will be important to subject the three differently structured small organic molecules to programs finding analogs with a higher affinity structure, while retaining the drug-like properties^27^. This is especially relevant for AF299 and ANO61, both of which lost their inhibitory efficacy in the presence of plasma, most likely by binding to plasma components. In contrast, ethopropazine at 10^−5^ M concentrations was still capable of reducing collagen-induced platelet aggregation in PRP and suppressing thrombus build-up in whole blood under flow.

Other laboratories have used well-plate assays aiming to find platelet-inhibitory molecules, based on light absorption, luminescence or fluorescence measurements, but so far only using 96 or 384 well-plate formats^35,36^. Those efforts have led to the finding of indole, diaminobenzene and imidazothiadiazole derivatives targeting the thrombin receptors PAR1 or PAR4^37–40^. Regarding GPVI, the current literature is restricted to an in silico screening of small molecules interfering with the ligand-binding domain^41^. To our knowledge, UHT-based screening methods in platelets have not yet been performed targeting the platelet Ca^2+^ signaling dynamics in the search for new agonist-selective signaling blockers.

Examination of the effects of 22 pharmacological agents interfering with platelet Ca^2+^ signaling, illustrated the major differences in the signaling domains downstream of GPVI- (CRP-induced) or PAR1/4 (thrombin-induced)^20,21^. These differences were supported by Reactome pathway analysis, the results of which can serve as a valuable starting point for further screening studies. For instance, it has been shown that cytokine and secondary mediator signaling, pathways overrepresented after GPVI activation, are carried out by platelets, thus reflecting a role of GPVI in inflammation and immune response^11,42^. On the other hand, the thrombin-induced signaling domain included a higher contribution of Rho GTPases, which in platelets are known to associate with the cytoskeleton^43,44^. Furthermore, the partially non-overlapping signaling domains from the Reactome pathway analysis can help to explain why PAR4 and GPVI seem to contribute more to arterial thrombosis than to hemostasis^2,45^. This hypothesis is supported by mouse knockout studies, indicating a thrombosis-stimulating role of these platelet receptors with limited effects on tail bleeding times^46^.

The workflow in the present study for initial compound prioritization, *i.e.* to limit the number of compounds for further testing in secondary assays, was based on stringent filtering criteria. This can be seen as a limitation because of the risk of filtering out false-negative compounds for instance due to rare robot-dependent injection leakages in some wells. Selection of non-fluorescence-interfering compounds for the diversity-based compound library may have also resulted in lower drop-off of compounds. The chosen ‘reproducibility’ difference index threshold of 30% can also be considered as stringent. Further, we discarded several compounds with unfavorable ADME-Tox characteristics, which after chemical modification could still give suitable anti-platelet agents. However, within the frame of the study, this selection was needed for obtaining a manageable number of compounds for follow-up experiments. With current improvements of computational methods to better identify potential clinical drugs, it is expected that future screening assays can be based on improved filtering techniques.

The identified compound ethopropazine from the so-called Prestwick chemical library of FDA-approved drugs. This library has more often been used to find novel functions of existing drugs and/or repurpose them, for instance in screening efforts for antibiotics or anti-viral compounds^25,26,47^. Ethopropazine was earlier commercialized for the treatment of Parkinson’s disease, based on its interaction with benzodiazepine receptors and butyryl cholinesterase in plasma^48,49^. Early literature also indicated that ethopropazine targets the M1/2 acetylcholine receptors and ionotropic glutamate receptors (Fig. S10a)^50,51^. These receptors are expressed in platelets at very low levels or not at all^52^. Thus, our finding points to a novel off-target effect of ethopropazine to affect platelet functions.

In 1980, it was reported that trifluoperazine, a related phenothiazine compound, inhibits platelet aggregation as a calmodulin antagonist^53,54^. Later it was suggested that phenothiazine analogs in platelets block Ca^2+^ uptake and cannot be selective inhibitors of calmodulin^55^. These early findings, which took place before the identification of platelet receptors and signaling pathways, are compatible with the present platelet inhibitory profile of ethopropazine, which now appears to be receptor and pathway-specific. Importantly, we also find that ethopropazine markedly reduces collagen-induced thrombus growth upon blood perfusion at high shear rate, *i.e.* an ex vivo method known to mimic in vivo arterial thrombosis mice models^46^.

Regarding the action mechanism of ethopropazine, the systematic comparison made with 22 known platelet inhibitors illustrated the broad signaling domain of pathways contributing to the CRP-induced Ca^2+^ response. Guided by the Reactome pathway analysis, we checked ethopropazine effects on platelet cAMP level, Ca^2+^ entry, secondary mediator release and protein tyrosine phosphorylation. This indicated that the most likely target of ethopropazine in GVPI-stimulated platelets is on the level of PI3K, such as reported before for inhibition of the isoforms PI3Kα^56^, PI3Kβ^29^ or PI3Kδ^34^.

For the compounds ANO61 and AF299, we observed low effectivity—likely due to high plasma binding—upon agonist-induced PRP aggregation. Plasma protein binding are known to affect the bioavailability and distribution of bioactive compounds, which effect partly determines their pharmacokinetics^57^. For instance, clinically used tyrosine kinase inhibitors are known to bind to plasma factors, which reduces the drug efficiency and limits diffusion through the cell membrane^58,59^. This may also be the case for AF299 and ANO61. In this respect, structure–activity studies and medicinal chemistry programs are essential for further compound development to improve specificity and reduce the risk of failure in clinical trials^60^.

Agents effectively preventing arterial thrombosis are still the subject of intense research. Biological screening approaches aim to produce a reasonable number of high-quality hits that can result in promising lead compounds and finally drug candidates. Here, we show the potential of the UHT-based measuring intracellular Ca^2+^ levels to discover antiplatelet hits. The present method is promising for future small-scale and large-scale screening purposes targeting platelet activation.

## Methods

Additional methods are provided in the Supplement.

### Blood collection

Blood was obtained by venipuncture from healthy volunteers, who had not received anti-platelet medication for at least two weeks and provided full informed consent according to the declaration of Helsinki. The study was approved by the Medical Ethics Committee of Maastricht University Medical Centre^+^ (NL31480.068.10). Blood was collected into 3.2% trisodium citrate after discarding the first 3 ml of blood (Vacuette tubes, Greiner Bio-One, Alphen a/d Rijn, The Netherlands).

For the small molecule screening, two bags of 330 mL platelet concentrates were obtained from Stichting Sanquin (Amsterdam, The Netherlands; permission NVT0505.01). These concentrates were pooled from five healthy donors with identical ABO and Rh(D) compatible blood types (11 × 10^9^ platelets/mL PAS-E plasma). The platelet batches were available after safety screening, at two days after blood drawing. Platelets were isolated immediately after receival and checked for full responsiveness to CRP and thrombin. Platelet count and hematological parameters were measured with an XN-9000 analyzer (Sysmex, Kobe, Japan).

### Small-molecule screening libraries

Compound libraries were screened at Pivot Park Screening Centre (PPSC, Oss, The Netherlands). Tested libraries were the Prestwick Chemical Library (1,280 compounds)^47^, divided over one 1,536-well plate, and a diversity-based SMC (Small Molecule Compound) library containing 15,355 small molecules, designed by Pivot Park Screening Centre, which were divided across other 1536-well plates. The SMC library was based on 15 molecular scaffolds, all obeying the Lipinski rule of five for orally active drugs, *i.e.* no more than 5 hydrogen bond donors (the total number of nitrogen–hydrogen and oxygen-hydrogen bonds); no more than 10 hydrogen bond acceptors (all nitrogen or oxygen atoms); a molecular mass > 500 Da; and a calculated octanol–water partition coefficient < 5^23,61^, in majority also passing the Ghose filter^62^. Physicochemical characteristics of compounds in the SMC library are provided in Fig. S2 and detailed in Datafile S1. All compounds were dissolved into DMSO and maintained at room temperature during the screening procedure. The compounds dissolved in DMSO were transferred to the 1536-well plates using an Echo acoustic dispenser (200 nL/well), and then diluted with Hepes buffer pH 7.45 (30 µL).

### Platelet isolation

Platelet-rich plasma (PRP) and washed platelets were isolated from citrate-anticoagulated human blood, basically as described^29^. In brief, PRP was prepared from citrated blood by centrifugation at 260 g for 15 min. For PRP measurements, the platelet count was adjusted to 250 × 10^9^/L using autologous platelet-free plasma. Washed platelets were prepared from PRP, supplemented with 1:10 acidic citrate dextrose (ACD; 80 mM trisodium citrate, 52 mM citric acid, and 180 mM glucose), by centrifugation at 2230 g for 2 min. The pelleted platelets were resuspended in Hepes buffer pH 6.6 (10 mM Hepes, 136 mM NaCl, 2.7 mM KCl, 2 mM MgCl_2_, 5.5 mM glucose and 0.1% bovine serum albumin), and after supplementation with 1:15 ACD and 0.1 U/mL apyrase, the centrifugation step was repeated. Washed platelets were resuspended in Hepes buffer pH 7.45 (10 mM Hepes, 126 mM NaCl, 2.7 mM KCl, 2 mM MgCl_2_, 5.5 mM glucose and 0.1% bovine serum albumin) at desired count.

### Small-molecule UHT-based screening of platelet Ca^2+^ responses in 1,536-well plate

For screening purposes, washed platelets (400 × 10^9^/L) were loaded with Calcium-6, as described previously^22^. Calcium-6 loaded platelets resuspended at 400 × 10^9^/L into Hepes buffer pH 7.45 plus 1 mM CaCl_2_ were distributed as 4 μL volumes over a 1536-well plate using a Certus dispenser device (Guger, AG, Gwatt, Switzerland). For the screening, a total of 16,635 small molecules were transferred to 13 source 1536-well-plates, according to a predefined layout using an Echo acoustic dispenser (200 nL/well). The molecules were then diluted with Hepes buffer pH 7.45 (30 µL). Per 1536-well plate, 192 wells were for agonist controls (vehicle), and 64 wells were for buffer controls (resting), the remaining 1280 wells were used for compounds. Compound plates were used four times, *i.e.* for duplicate screening with either agonist, unless indicated otherwise. Screening of subsets of compounds was done in one day using the same platelet pool.

The Calcium-6 loaded platelets divided over wells were pre-incubated (5 min) by automated pipetting with individual compounds (2 µL, 10 µM, f.c.) or vehicle solution. The pipetting, fluorescence measurements and agonist additions were performed at 37 °C with an automated FLIPR-Tetra machine (Molecular Devices, San Jose, CA, USA). All platelet, compound and agonist plates were placed inside the FLIPR robot. As agonists, CRP (2 µL, 10 µg/mL, f.c.) or thrombin (2 µL, 4 nM, f.c.) were used, which were dissolved into Hepes buffer pH 7.45. Calcium-6 fluorescence changes were measured with CRP for 10 min, and with thrombin for 5 min. The measurements were performed in the presence of 1 mM CaCl_2_ using the FLIPR-Tetra machine and taking into account the length of the Ca^2+^ signal^22^. Calcium-6 fluorescence was measured in all wells simultaneously at excitation and emission wavelengths of 485 nm and 525 nm, respectively.

Of note, the UHT-based screening assay in 1,536-well plate format was performed by robot injection of 2 µL of agonist or vehicle solution into 6 µL of dye-loaded platelets plus compound per well. Injection occurred by temporarily inserting a polypropylene tip (one per well) into the cell suspension, controlled by the FLIPR-Tetra machine. The injection speed was optimized to achieve optimal mixing, but the injection of a relatively large volume of agonist solution as well as light pathway interference of the tip resulted in a drop in fluorescence.

### Cytosolic Ca^2+^ responses of Calcium-6 loaded platelets in 96-well plate

Washed human platelets (400 × 10^9^/L) were loaded with Calcium-6 solution (1:1 vol./vol) for 2 h at room temperature. After the washing step, the Calcium-6 loaded platelets (200 × 10^9^/L) were preincubated with known inhibitors or selected screening compounds for 10 min at room temperature. The platelets in Hepes buffer pH 7.45 with 1 mM CaCl_2_ or 0.1 mM EGTA were distributed over a 96-well plate (200 μL). Changes in Calcium-6 fluorescence were measured, upon stimulation with CRP (5–10 µg/mL, f.c.) or thrombin (2–4 nM, f.c.) by automated pipetting in a FlexStation 3 machine (Molecular Devices, San Jose, CA, USA). Calcium-6 fluorescence was continuously measured per row, at excitation and emission wavelengths of 485 nm and 525 nm, respectively. Duplicate wells were used per condition. Mean values of effects of inhibitors were log2 transformed for heatmap visualization.

### Automated Ca^2+^ curve analysis and filtering of screening assay

The Calcium-6 fluorescence curves were analyzed per well (96- or 1536-well plates) using a custom-made algorithm written in Matlab (MathWorks, Natick, MA, USA). Seven curve characteristics were defined per trace (*P1-7*). The characteristics were converted into the following parameters: *P1*, baseline level before agonist; *P2*, maximal Ca^2+^ increase; *P3*, slope 1; *P4*, peak fluorescence after agonist addition; *P5-P6*, slopes 2 and 3; *P7*, area under the curve (AUC), the latter one indicating a cumulative agonist effect. The slopes 1–3 represent the rates of fluorescence signal changes by agonist addition per curve section. Slope 1 refers to the initial signal increase. Slopes 2 and 3 to a subsequent decrease and a secondary increase after the peak level, respectively. Slope 2 was equaled 0 if absent. Slope 3 was calculated from t = 650 s for CRP datasets, and from t = 500 s for thrombin datasets. The Matlab script for obtaining all curve parameters is accessible via https://github.com/sobotav/CaCurveAnalysis (see also the Supplemental Information).

Due to residual increases in fluorescence baseline before agonist addition (caused by dye leakage), for response calculations, *P1* immediately before agonist addition was taken was basal F_0_ fluorescence, both in 96-well and 1,536-well plate formats. Subsequent platelet response expression as F – F_0_ appeared to be a more robust manner than using ratio values of F/F_0_. Parameter values were normalized to the control conditions per well plate, and then Z-score was calculated per parameter and plate, as the number of standard deviations above or below the mean^63^.

The small molecule screening was performed in duplicate plates (A and B), the results of which were used for calculation of the difference index (DI), as a factor determining the variability between duplicate conditions across two well plates. This parameter was calculated as the ratio between the maximal signal difference and the two signal values: DI = *(abs (A–B))/(max AB–min AB)*. Herein, *max AB–min AB* indicates the difference of both fluorescence values. In other words, the relative difference in signal was calculated from the absolute difference between two signals divided by the total range (maximum–minimum) of values of both signals. The DI is defined as the 95^th^ percentile of the calculated relative difference. Based on sensitivity analysis, a DI threshold of 30% was chosen as optimal, meaning that 95% of the relative difference between signals did not exceed 30%. Originally screened were 16,635 compounds with either agonist, CRP or thrombin. Applying the difference index (DI) to correct for high variability between duplicates, led to 11,883 and 12,952 compounds for CRP and thrombin, respectively, for subsequent analysis. Selected curves were normalized to the means of control agonist wells (set at 100%) per plate. For compound wells, Z-scores were calculated per parameter *P1-7* and per plate. Compounds with a Z-score >| 4 | were considered to be active and was used to filter compounds with an assumed relevant effect.

Median values before and after the addition of drug were used to identify compounds with a signal- or platelet-interfering effect (*i.e.*, autofluorescence, fluorescence quenching, or dye leakage). Fluorescence curves were inspected for the correct addition of agonist. An ADME-Tox filtering tool available at https://fafdrugs4.rpbs.univ-paris-diderot.fr was used to remove compounds with assumed detrimental metabolic or toxicity characteristics, based on in silico predictions. For the re-screening, selected compounds were exposed to Calcium-6 loaded platelets at 10 µM in 96-well plates using a FlexStation 3 robot. Compound inhibition of > 25% was taken as a filter for continued experimenting at further stages. Dose–response data were fitted by least squares regression analysis, which provided IC_50_ values.

### Light transmission aggregometry

Isolated platelets in Hepes buffer pH 7.45 or in PRP (250 × 10^9^/L) were pre-incubated with indicated compound or vehicle for 5 min at room temperature and 5 min at 37 ºC. Platelet aggregation was then recorded under stirring (1200 rpm) using a Chronolog optical aggregometer (Havertown, PA, USA) at 37 °C^30^. Aggregation was induced by collagen type I (1 µg/mL), CRP (10 µg/mL), thrombin (1 or 4 nM), or TRAP6 (15 µM), as indicated in the figure legends. Curves of light transmission changes were analyzed for maximum aggregation of 8 min after the addition of the agonist.

### Microfluidic whole blood thrombus formation

Whole blood thrombus formation was measured as a multiparameter assay using parallel-plate microfluidic chambers, as described before^31^. Rectangular glass coverslips were coated with three microspots (0.5 µL per spot). The adjacent microspots consisted of: *(i)* 100 µg/mL collagen type I (microspot M1), *(ii)* 30 µg/mL collagen type I (M2), and *(iii)* 100 µg/mL collagen III (M3). Per flow run, 500 µL of citrated blood was anticoagulated with PPACK (40 µM) and recalcified directly before perfusion. The most active microspot (collagen I) was mounted at the downstream side of the flow chamber^31^. Blood flow was for 3.5 min at a wall-shear rate of 1000 s^−1^. The formed thrombi were post-perfused with rinse buffer (10 mM Hepes, 136 mM NaCl, 2.7 mM KCl, 2 mM MgCl_2_, 2 mM CaCl_2_, 5.5 mM glucose, 0.1% bovine serum albumin, and 1 U/mL heparin, pH 7.45), to which were added three fluorescent labels (FITC-labeled anti-fibrinogen mAb 10 μg/mL, AF568 annexin A5 5 μg/mL, and AF647 anti-CD62P mAb 8 μg/mL). Unbound label was removed by 2 min perfusion with rinse buffer. Brightfield images (during staining) and fluorescence images (after rinse) were taken under flow with an EVOS-FL microscope (Life Technologies, Bleiswijk, The Netherlands), equipped with Cy5, RFP and GFP LEDs, an Olympus UPLSAP 60 × oil-immersion objective, and a 1360 × 1,024 pixel CCD camera^64^. Per condition, flow runs were performed at least in duplicate. Semi-automated scripts were used for image analyses, as described in the Supplement.

### Reactome pathway analysis

A panel of 22 reference compounds inhibiting platelet functions (see Table S1) were linked to their target proteins using UniProtKD assignments. Target proteins for inhibitors that significantly affected agonist-induced [Ca^2+^]_i_ traces for at least 3 parameters (one-way ANOVA corrected for multiple comparisons by a Holm-Sidak test) were uploaded into Reactome to find the corresponding pathways. The listed protein identifiers (as gene symbols) were screened for pathways with n ≥ 4 entities and false discovery rates (FDR) of < 1%. Primary Reactome data are provided in Datafile S1.

### Data and statistical analyses

The data are shown as means ± standard deviation (SD). Graphpad Prism 9 software (version 9.4.1, La Jolla, CA, USA) was used for statistical analysis. Normality was evaluated using the Shapiro–Wilk test, which determined the choice of parametric or nonparametric testing. Statistical significance was set at *p* < 0.05. The package R version 3.2.5 (www.r-project.org) was used for correlation analyses, computation of k-means clustering, decision on number of clusters, Euclidean distance matrix, and to generate heatmaps.

### Supplementary Information

Supplementary Information 1.

Supplementary Information 2.

Supplementary Figure S9.

## Data Availability

The data that support the findings of this study are included in this article and its supplementary information files. The Matlab algorithm has been deposited on GitHub (https://github.com/sobotav/CaCurveAnalysis). Indirect data are available from the corresponding author upon reasonable request.

## Abbreviations

BSA: Bovine serum albumin
CRP: Collagen-related peptide
FDR: False discovery rate
FITC: Fluorescein isothiocyanate
GPVI: Glycoprotein VI
PI3K: Phosphoinositide 3-kinase
PLC: Phospholipase C
PRP: Platelet-rich plasma
TRAP6: Thrombin receptor-activating peptide 6
