# Supplementary material for: Ultra-high throughput-based screening for the discovery of antiplatelet drugs affecting receptor dependent calcium signaling dynamics
Source: Sci Rep. 2024 Mar 14;14:6229. doi: 10.1038/s41598-024-56799-4 (PMC10940705; doi:10.1038/s41598-024-56799-4)
Supplement: Supplementary file 2 — Supplementary Information 2. [file 41598_2024_56799_MOESM2_ESM.pdf]

Supporting information

Ultra-high throughput-based screening for the discovery of antiplatelet drugs affecting receptor dependent calcium signaling dynamics

Delia I. Fernández,[1,2] Sara Troitiño,[2#] Vladimír Sobota,[3,4#] Bibian M. E. Tullemans,[1,5] Jinmi Zou,[1,5] Helma van den Hurk,[6] Ángel García,[2] Saman Hornanejad,[6] Marijke J. E. Kuijpers,[1,7]* Johan W. M. Heemskerk[1,5]*

From: [1]The Department of Biochemistry, CARIM, Maastricht University, 6229 ER Maastricht, The Netherlands; [2]Platelet Proteomics Group, CiMUS, Universidade de Santiago de Compostela, 15782 Santiago de Compostela, Spain; [3]IHU-LIRYC, Electrophysiology and Heart Modeling Institute, Fondation Bordeaux Université, 33604 Bordeaux, France; [4]University of Bordeaux, Institut de Mathématiques de Bordeaux, UMR5251, 33 405 Talence, France; [5]Synapse Research Institute, Kon. Emmaplein 7, 6217 KD, Maastricht, The Netherlands;[6]Pivot Park Screening Centre, 5349 AB Oss, The Netherlands; [7]Thrombosis Expertise Centre, Heart and Vascular Centre, Maastricht University Medical Centre+, 6229 HX, Maastricht, The Netherlands

[#]equal contribution

Supplementary materials and methods

Materials

Bovine serum albumin (BSA), D(+)-glucose, unfractionated heparin, apyrase, human placenta-derived collagen III (C4407), phorbol myristate acetate, 2-aminoetyl diphenylborinate (2-APB), DAG-kinase inhibitor I, DAG-kinase inhibitor II, GFX109203X, 3-isobutyl-1-methyl-xanthene (IBMX), and compounds PP2 and PP3 were purchased from Sigma-Aldrich (Zwijndrecht, The Netherlands). Collagen type I Horm was from Takeda (Hoofddorp, The Netherlands). Collagen-related peptide-crosslinked (CRP) was obtained from CambCol Laboratories (Cambridge, UK). Thrombin came from Enzyme Research Laboratories (South Bend, IN, USA); thrombin receptor-

activating peptide 6 (TRAP6) from Bachem (Bubendorf, Switzerland); D-phenylalanyl-prolyl-arginyl chloromethyl ketone (PPACK) from Santa Cruz Biotechnology (Dallas, TX, USA). Fluorescein isothiocyanate (FITC)-conjugated anti-fibrinogen mAb was purchased from DAKO (F0111; Santa Clara, CA, USA). Alexa Fluor (AF)568-conjugated annexin A5 came from Life Technologies (New York, NY, USA). AF657-labeled mouse anti-human CD62P mAb (Clone AK4) was from Biolegend (London, UK). Calcein-AM and Pluronic F-127 were from Molecular Probes (Leiden, The Netherlands). The FLIPR Calcium-6 assay kit was from Molecular Devices (San Jose, CA, USA). Small molecules obtained as positive hits in the high-throughput screening were obtained from Specs (Zoetermeer, The Netherlands) with acceptable purity levels (>95%). Indomethacin was from AstraZeneca (The Hague, The Netherlands). Eptifibatide was from GlaxoSmithKline (Greenford, UK). Aprotinin, leupeptin, Na_3VO_4, pepstatin A, and the inhibitors PD-98059, Ro-318220, rottlerin, PKCβ inhibitor (3-(1-(3-imidazol-1-ylpropyl)-1H-indol-3-yl)-4-anilino-1H-pyrrole-2,5-dione) and LY294002 were from Calbiochem (La Jolla, CA, USA). PKCθ inhibitor was a kind gift from Boehringer Ingelheim Pharmaceuticals[1]. Ethopropazine hydrochloride, NSC87877, and SHP099 were obtained from Cayman Chemicals (Ann Arbor, MI, USA). Inhibitors of PI3K isoforms, PIK-75 and TGX-221, came from Baker (Melbourne, Victoria, Australia). Protein kinase A inhibitor H89 was from Alexis (Läufelfingen, Switzerland). Thapsigargin and PRT-060318, 2-((1R,2S)-2-aminocyclohexylamino)-4-(m-tolylamino) pyrimidine-5-carboxamide, were from Bio-Connect (Huissen, The Netherlands). All compounds from commercial suppliers were of analytical grade. Antibodies for western blotting were monoclonal phosphotyrosine antibody (clone 4G10, 05-321, Millipore), rabbit anti-human p-PLCγ2 (Y^{759}, Mab7277, R&D Systems); mouse anti-PLCγ2 (sc-5283, Santa Cruz Biotechnology); rabbit anti-p-Syk (Y^{525} + Y^{526}, ab58575, Abcam); mouse anti-Syk (sc-1240, Santa Cruz); or GAPDH (Sigma, G9545).

Flow cytometric platelet viability assay

Washed platelets (50 × 10^9/L) in Hepes buffer pH 7.45 were pre-incubated for 10 min at room temperature with selected compounds or vehicle control (<0.5% DMSO, f.c.). Subsequently, the platelets were loaded with calcein-AM (20 μM) for 20 min at 37 ºC. The de-esterified calcein that is accumulated and retained into the cytosol is a common viability marker[2]. Triplicate samples of the calcein-loaded platelets were measured for mean fluorescence intensity, using an Accuri C6

flow cytometer (BD Bioscience, Franklin Lakes, NJ, USA). Triplicate samples per condition were analyzed. The threshold level for non-toxicity wase set at 10% DMSO.

Western blotting

Washed platelets (500 × 10^9/L) were pre-incubated at room temperature with ethopropazine (32 or 10 µM, 10 min) or vehicle (DMSO < 0.5%), in presence of eptifibatide (9 µM) to avoid platelet aggregation. The platelet samples were heated for 4 min at 37 ºC in a Chronolog aggregometer. After stirring for 1 min, activations were performed for 90 s with CRP (5 µg/mL) and thrombin (4 nM). Lysates were obtained by adding 4× ice-cold lysis buffer (600 mM NaCl, 40 mM Tris, 4 mM EGTA, 4 mM EDTA, and 4% NP-40, pH 7.5) plus 10 mM Na_3VO_4, 4 mM PMSF, 5 mg/mL leupeptin, 20 µg/mL aprotinin, and 5 µg/mL pepstatin A. Lysates were resuspended in sample buffer and boiled. Proteins were separated by SDS 11% polyacrylamide gel electrophoresis, followed by immunoblotting using polyvinylidene difluoride membranes. After blocking with 5% BSA in TBS-T (20 mM Tris-HCl pH 7.6, 150 mM NaCl, 0.1% Tween 20), the membranes were incubated using a primary antibody, as stated. The immune-stained membranes were treated with horseradish peroxidase-labeled goat anti-mouse antibody (31430, dilution 1/5000) or goat anti-rabbit antibody (31460, Pierce, Rockford, IL, USA). The membranes were then washed and processed using an enhanced chemiluminescence system (ECL, Pierce, Rockford, USA).

Parameters of microscopic image analysis

Microscopic images were analyzed in Fiji (version 2.0.0) using semi-automated scripts, which allow manual threshold resetting. Detailed description of the method is provided in the supplement of Ref.[3]. Scoring of brightfield images was based on a predefined image gallery[3]. Outcome parameters for brightfield images were: *P1*, thrombus morphological score (scale 0-5); *P2*, platelet surface area coverage (SAC%); *P3*, thrombus contraction score (scale 0-3); *P4*, thrombus multilayer score (scale 0-3), and *P5*, platelet multilayer coverage (SAC%). Parameters obtained from fluorescence images were *P6*, phosphatidylserine exposure (AF568-annexin A5, SAC%); *P7*, P-selectin expression (AF647 anti-CD62P mAb, SAC%); and *P8*, integrin αIIbβ3 activation (FITC anti-fibrinogen mAb, SAC%). Mean values per condition and parameter were calculated, and data were univariate scale 0-10 across all surfaces. For subtraction heatmaps, vehicle values were subtracted per condition[4].

Matlab script for automated analysis of Calcium-6 fluorescence curves

The repository contains Matlab scripts with implementation of a method for automated analysis of fluorescence curves obtained from human Calcium-6-loaded platelets. The method performance is demonstrated on four sets of fluorescence curves. The software is licensed under the GNU GPL v3. For data analysis, the fluorescence curves were obtained from 96 and 1,536-well plate assays with human Calcium-6-loaded platelets. In the 96-well plate condition, a tested compound was pre-incubated, implying that the fluorescence curve shows only the response to the agonist (CRP or thrombin). In the 1,536-well plate condition, the tested compound was added first, followed by the agonist addition. Since the fluorescent curves are slightly different for each type of the well plate, they are analyzed by two distinct functions: analyzeData_96.m for the 96-well plate and analyzeData_1536.m for the 1,536-well plate. The script is uploaded at: https://github.com/sobotav/CaCurveAnalysis.

Supplemental datafile S1

Sheet A. Reference inhibitors, data and statistics.

Sheet B. Reference inhibitors: UniProt assignments.

Sheet C. Reactome pathways and statistics.

Sheet D. Whole blood microfluidic flow assay.

Sheet E. Raw small molecule screening data (CRP).

Sheet F: Raw small molecule screening data (thrombin).

Supplemental datafile S2

Original uncropped images of gels from Figure S9d.

References

1 Gilio, K. *et al.* Functional divergence of platelet protein kinase C (PKC) isoforms in thrombus formation on collagen. *J Biol Chem* **285**, 23410-23419 (2010).

2 Hartley, P. S., Savill, J. & Brown, S. B. The death of human platelets during incubation in citrated plasma involves shedding of CD42b and aggregation of dead platelets. *Thromb Haemost* **95**, 100-106 (2006).

3 Huang, J. *et al.* Roles of focal adhesion kinase PTK2 and integrin αIIbβ3 signaling in collagen- and GPVI-dependent thrombus formation under shear. *Int J Mol Sci* **23**, 8688 (2022).

4 Van Geffen, J. P. *et al.* High-throughput elucidation of thrombus formation reveals sources of platelet function variability. *Haematologica* **104**, 1256-1267 (2019).

5 Li, Z. *et al.* An important role of the SRC family kinase Lyn in stimulating platelet granule secretion. *J Biol Chem* **285**, 12559-12570 (2010).

6 Getz, T. M., Manne, B. K., Buitrago, L., Mao, Y. & Kunapuli, S. P. Dextran sulphate induces fibrinogen receptor activation through a novel Syk-independent PI-3 kinase-mediated tyrosine kinase pathway in platelets. *Thromb Haemost* **109**, 1131-1140 (2013).

7 Badolia, R., Kostyak, J. C., Dangelmaier, C. & Kunapuli, S. P. Syk activity is dispensable for platelet GPIb-IX-V signaling. *Int J Mol Sci* **18**, 1238 (2017).

8 Reilly, M. P. *et al.* PRT-060318, a novel Syk inhibitor, prevents heparin-induced thrombocytopenia and thrombosis in a transgenic mouse model. *Blood* **117**, 2241-2246 (2011).

9 Gilio, K. *et al.* Non-redundant roles of phosphoinositide 3-kinase isoforms alpha and beta in glycoprotein VI-induced platelet signaling and thrombus formation. *J Biol Chem* **284**, 33750-33762 (2009).

10 Kim, S., Garcia, A., Jackson, S. P. & Kunapuli, S. P. Insulin-like growth factor-1 regulates platelet activation through PI3-K alpha isoform. *Blood* **110**, 4206-4213 (2007).

11 Schmidt, E. M. *et al.* Ion channels in the regulation of platelet migration. *Biochem Biophys Res Commun* **415**, 54-60 (2011).

12 Dobrydneva, Y. *et al.* 2-aminoethoxydiphenyl borate as a prototype drug for a group of structurally related calcium channel blockers in human platelets. *Mol Pharmacol* **69**, 247-256, (2006).

13 Heemskerk, J. W., Feijge, M. A., Sage, S. O. & Farndale, R. W. Human platelet activation is inhibited upstream of the activation of phospholipase A_2 by U73343. *Biochem Pharmacol* **53**, 1257-1262 (1997).

14 Feijge, M. A. *et al.* Inter-individual variability in Ca^{2+} signalling in platelets from healthy volunteers: effects of aspirin and relationship with expression of endomembrane Ca^{2+}-ATPases. *Br J Haematol* **102**, 850-859 (1998).

15 Harper, M. T., Mason, M. J., Sage, S. O. & Harper, A. G. Phorbol ester-evoked Ca^{2+} signaling in human platelets is via autocrine activation of $P2X_1$ receptors, not a novel non-capacitative Ca^{2+} entry. *J Thromb Haemost* **8**, 1604-1613 (2010).

16 Lever, R. A., Hussain, A., Sun, B. B., Sage, S. O. & Harper, A. G. Conventional protein kinase C isoforms differentially regulate ADP- and thrombin-evoked Ca^{2+} signalling in human platelets. *Cell Calcium* **58**, 577-588 (2015).

17 Guidetti, G. F. *et al.* Phosphorylation of the guanine-nucleotide-exchange factor CalDAG-GEFI by protein kinase A regulates Ca^{2+}-dependent activation of platelet Rap1b GTPase. *Biochem J* **453**, 115-123 (2013).

18 Konopatskaya, O. *et al.* Protein kinase C mediates platelet secretion and thrombus formation through protein kinase D2. *Blood* **118**, 416-424 (2011).
19 Sakane, F., Hoshino, F., Ebina, M., Sakai, H. & Takahashi, D. The roles of diacylglycerol kinase alpha in cancer cell proliferation and apoptosis. *Cancers (Basel)* **13**, 5190 (2021).
20 Kassouf, N. *et al.* Phosphatidylinositol-3,4,5-trisphosphate stimulates Ca^{2+} elevation and Akt phosphorylation to constitute a major mechanism of thromboxane A_2 formation in human platelets. *Cell Signal* **27**, 1488-1498 (2015).
21 Flamm, M. H. *et al.* Multiscale prediction of patient-specific platelet function under flow. *Blood* **120**, 190-198 (2012).
22 Borsch-Haubold, A. G., Pasquet, S. & Watson, S. P. Direct inhibition of cyclooxygenase-1 and -2 by the kinase inhibitors SB 203580 and PD 98059. *J Biol Chem* **273**, 28766-28772, (1998).
23 Shah, B. H. *et al.* Molecular mechanisms involved in human platelet aggregation by synergistic interaction of platelet-activating factor and 5-hydroxytryptamine. *Exp Mol Med* **33**, 226-233 (2001).
24 Sage, S. O. & Rink, T. J. Inhibition by forskolin of cytosolic calcium rise, shape change and aggregation in Quin2-loaded human platelets. *FEBS Lett* **188**, 135-140 (1985).
25 Ma, P. *et al.* A newly identified complex of spinophilin and the tyrosine phosphatase, SHP-1, modulates platelet activation by regulating G protein-dependent signaling. *Blood* **119**, 1935-1945 (2012).
26 Bellio, M. *et al.* Catalytic dysregulation of SHP2 leading to Noonan syndromes affects platelet signaling and functions. *Blood* **134**, 2304-2317 (2019).

Supplemental figures

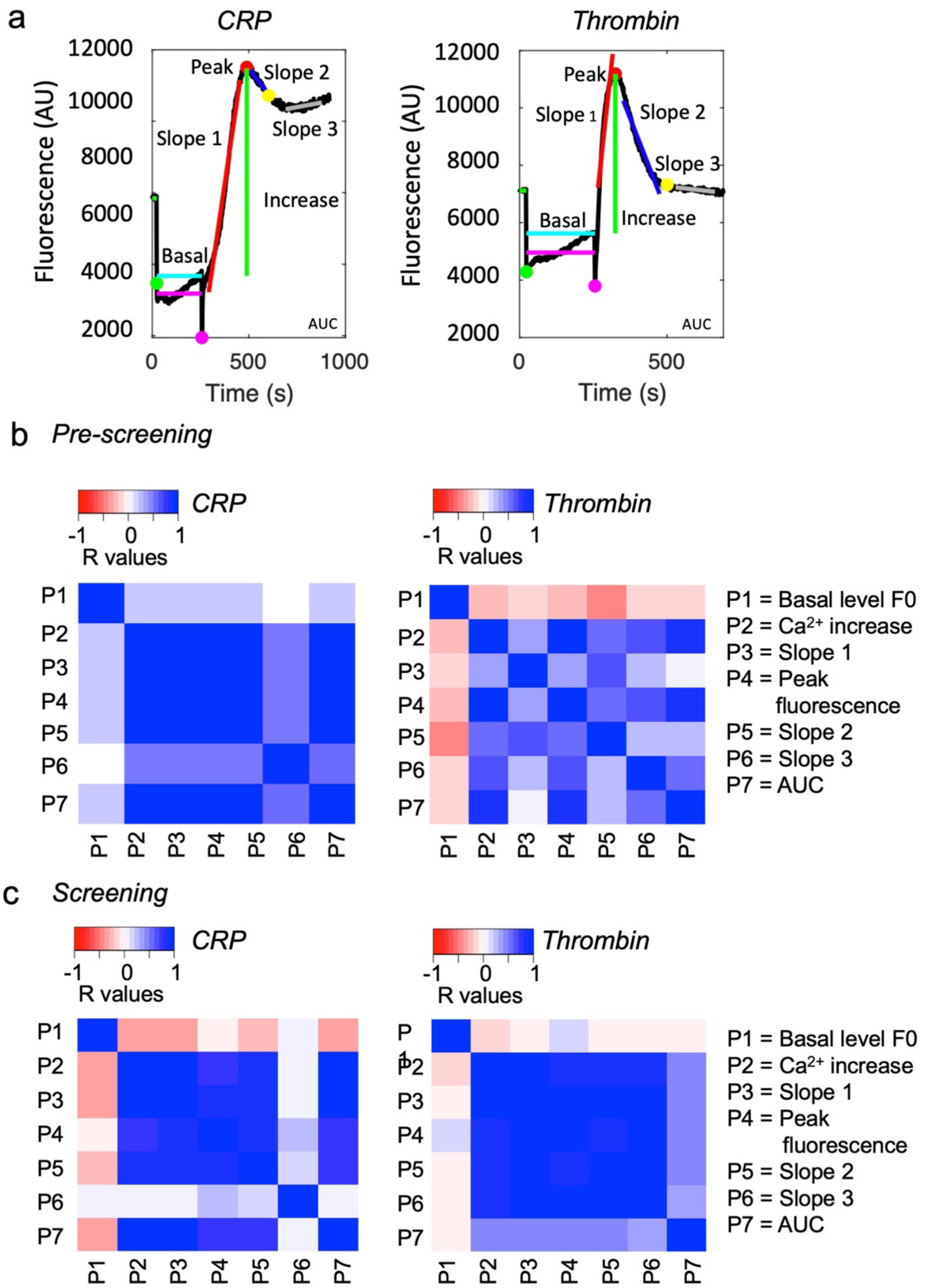

Figure S1. Ultrahigh throughput cytosolic Ca^{2+} trace profiling. *(a)* Representative fluorescence rises of Calcium-6 loaded platelets induced by CRP or thrombin in 1,536-well plates, recorded by FLIPR-Tetra. Color index: green dot = compound addition; purple dot = agonist addition; green line = median level before compound addition; purple line = median basal level before agonist addition (parameter *P1*); red dot = peak level (*P4*); red line = interpolation of slope 1 (*P3*); blue line = interpolation of slope 2 (*P5*); grey line = interpolation of slope 3 (*P6*); cyan line = fluorescence between addition of compound and agonist as a reference. Parameters area under the curve AUC (*P7*), and maximal cytosolic Ca^{2+} increase (*P2*) relative to the basal level. *(b-c)*, Calcium-6 loaded platelets in 96- or 1,536-well plates were stimulated with CRP (10 μg/mL) or thrombin (4 nM). The platelets in wells were preincubated with one of 22 inhibitors (pre-screening) or 16,635 small molecules (screening), as indicated. Shown are Spearman correlation matrices for the various curve parameters per agonist (CRP or thrombin) and per (pre)screening dataset. *(b)* Comparison of effects of 22 reference compounds in 96-well plate. *(c)*, Comparison of effects of 16,635 small molecules in 1536-well plates. Red and blue colors indicate negative and positive correlations, respectively.

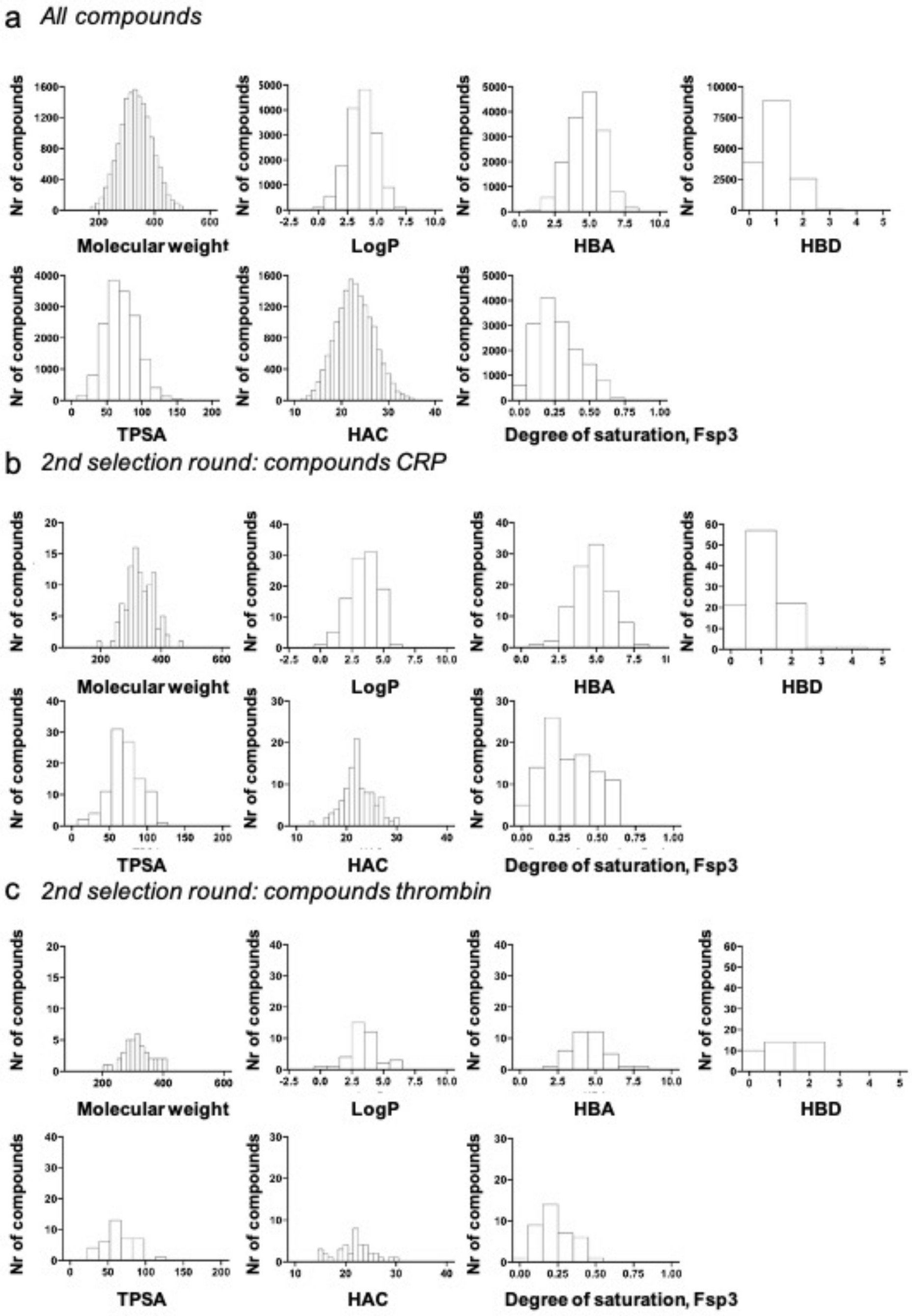

Figure S2. Physicochemical characteristics of compounds in the SMC library and of the selected effective compounds. Shown are physicochemical characteristics in the employed SMC library of 15,355 small molecule compounds (*a*) of 102 selected compounds for CRP-stimulated platelets (*b*) and of 38 compounds for thrombin-stimulated platelets (*c*) (2nd selection round). Characteristics are indicated of molecular weight; lipophilic partition coefficient (LogP); numbers of hydrogen bond acceptors (HBA) and donors (HBD); topological polar surface area of nitrogen, oxygen, phosphate and sulfur atoms (TPSA); heavy atom count (HAC) for total number of non-hydrogen atoms; and degree of carbon saturation (Fsp3).

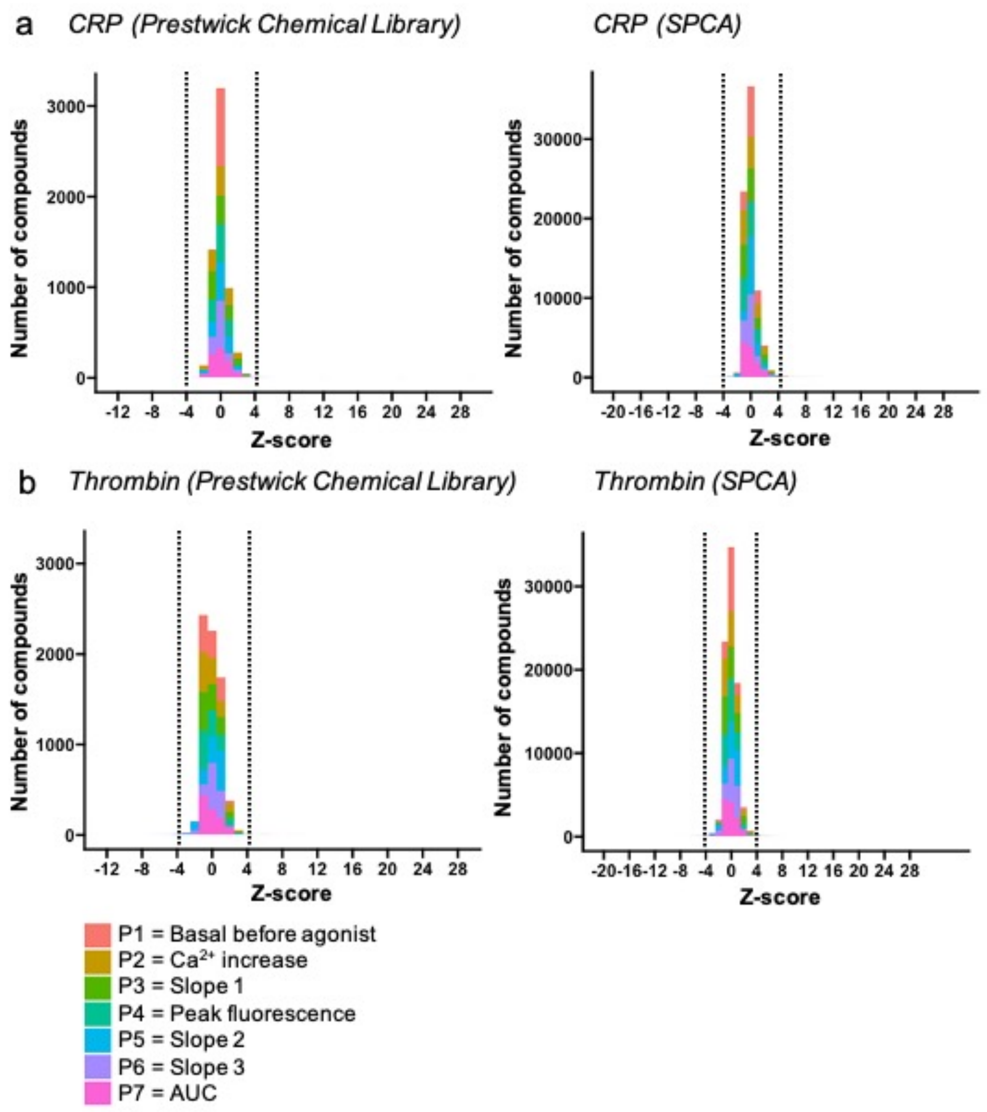

Figure S3. Distribution profiles of summed Z-scores per compound library and per agonist. The Prestwick Chemical library and the larger SMC library of small molecules were screened on effects of platelets stimulated with CRP (*a*) or thrombin (*b*). Seven parameters (*P1-7*) were derived from the cytosolic Ca^{2+} traces. Shown are stacked histograms of Z-scores for each parameter and compound. Dashed lines indicate Z-score threshold of > | 4 |.

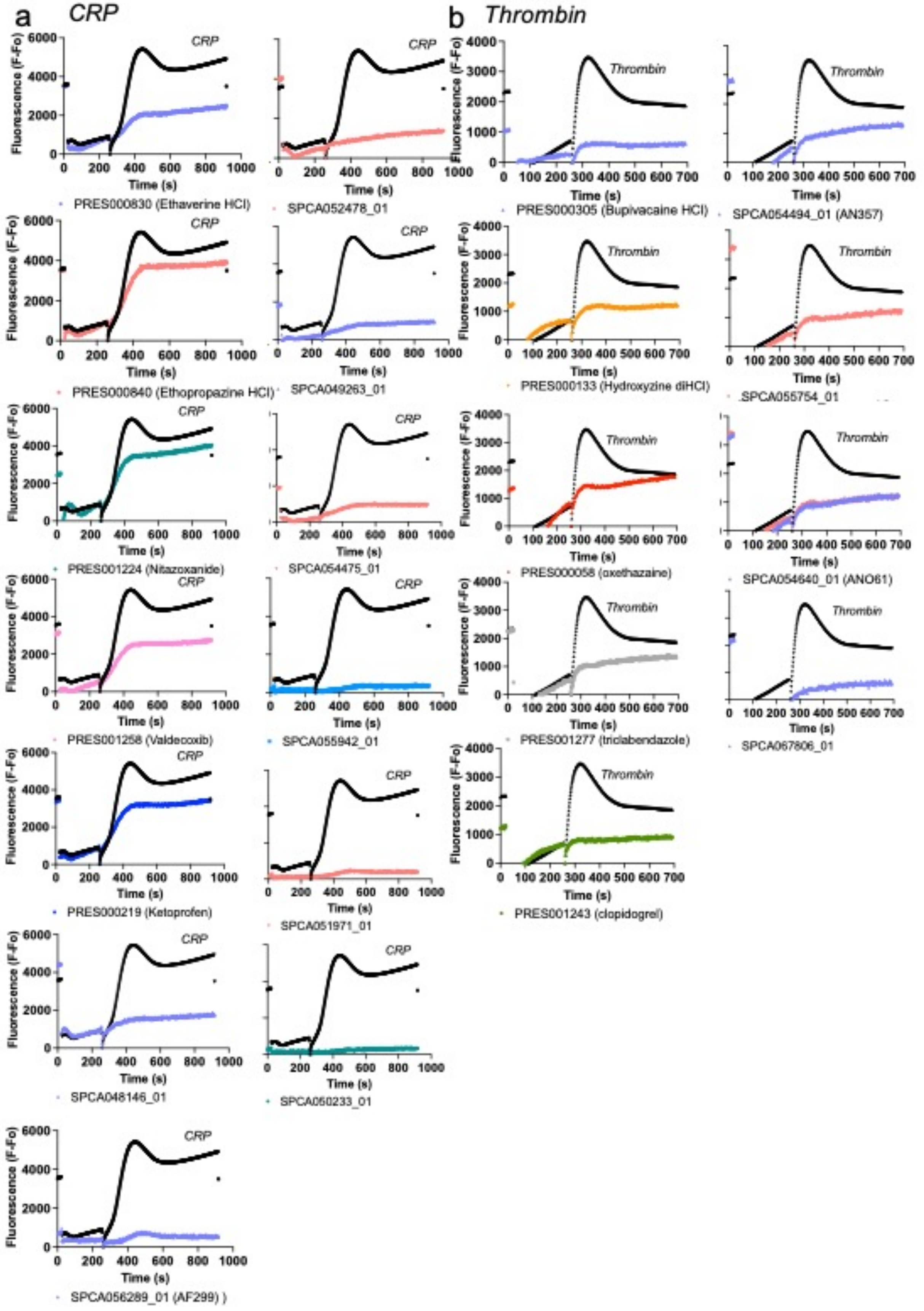
a CRP
b Thrombin
Fluorescence (F-Fo)
Time (s)
CRP
Thrombin
PRES000830 (Ethaverine HCl)
SPCA052478_01
PRES000840 (Ethopropazine HCl)
SPCA049263_01
PRES001224 (Nitazoxanide)
SPCA054475_01
PRES001258 (Valdecoxib)
SPCA055942_01
PRES000219 (Ketoprofen)
SPCA051971_01
SPCA048146_01
SPCA050233_01
SPCA056289_01 (AF299))
PRES000305 (Bupivacaine HCl)
SPCA054494_01 (AN357)
PRES000133 (Hydroxyzine diHCl)
SPCA055754_01
PRES000058 (oxethazaine)
SPCA054640_01 (ANO61)
PRES001277 (triclabendazole)
SPCA067806_01
PRES001243 (clopidogrel)

Figure S4. Platelet Calcium-6 fluorescence traces with selected small molecules during initial screening. Calcium-6 loaded platelets (4 µL, 400 × 10^9/L) in 1536-well plates were stimulated with CRP 10 µg/mL (*a*) or thrombin 4 nM (*b*) after preincubation with indicated compound (10 µM). Fluorescence changes from wells were recorded using a FLIPR-Tetra machine. Baseline fluorescence immediately before agonist addition was defined as F_0. Of note: Optimal mixing was achieved by the injection speed, but the injection of a relatively large volume of agonist solution as well as light pathway interference of the tip resulted in a drop in fluorescence.

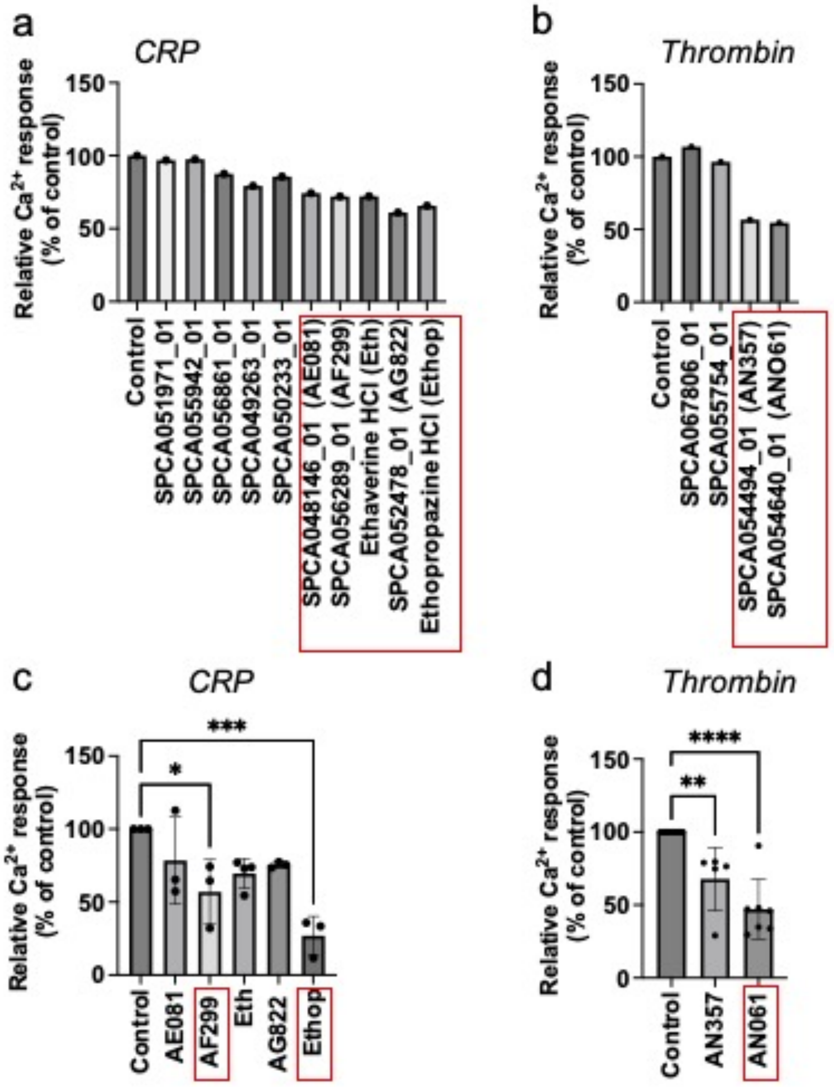

Figure S5. Re-assessment of first selected compounds. Calcium-6 loaded platelets (200 µL, 200 × 10^9/L) in 96-well plates were stimulated with CRP (10 µg/mL) or thrombin (4 nM), after 10 min preincubation with vehicle control medium (DMSO) or indicated concentrations (10 µM) of compounds passing 3 selection rounds. *(a-b)* Selected compounds were tested at 10 µM. (*c-d*) When inhibition was observed (red boxes), compounds were also tested at 30 µM to observe a dose-response. Data are presented as percentages of the maximal fluorescence signal. Mean ± SD (n = 3-7 donors), **P*<0.05, ***P*<0.01, ****P*<0.001, *****P*<0.0001 vs. vehicle, one-way ANOVA with Tukey post-hoc test.

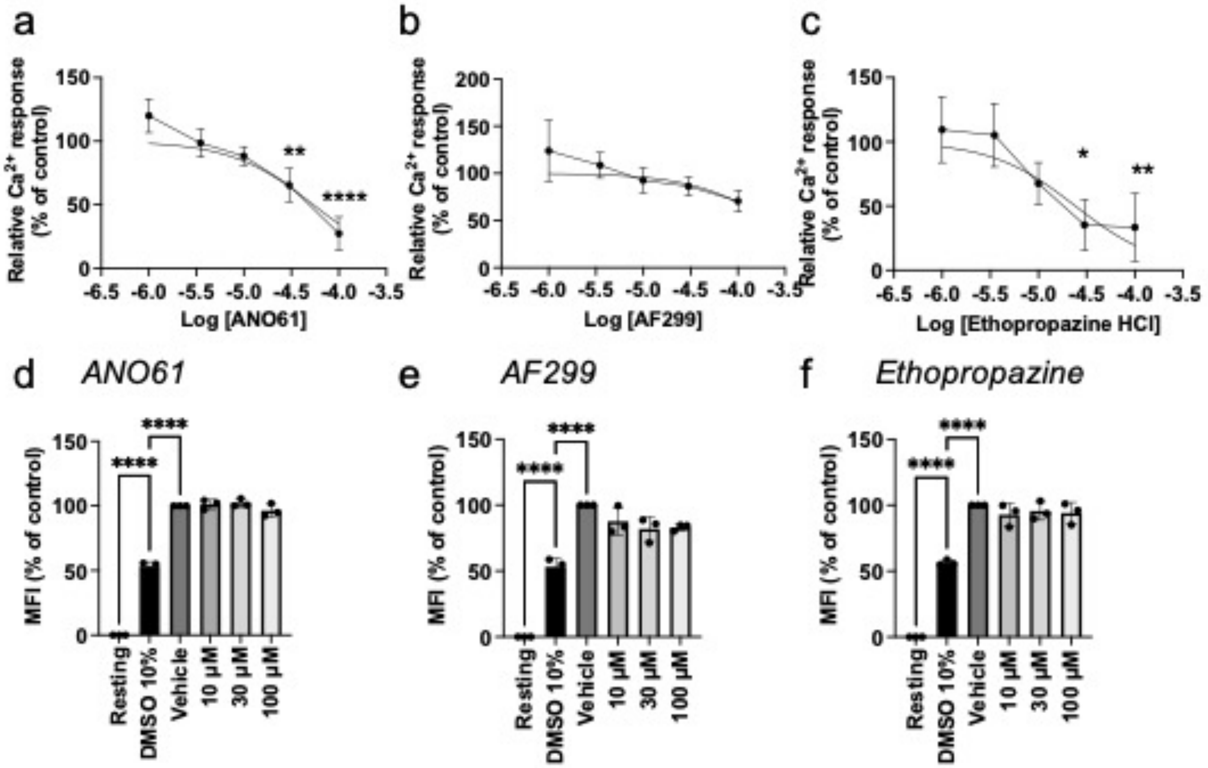

Figure S6. Toxicity and bioavailability of three hit compounds. (*a-c*), Dose-response effects of ANO61, AF299 and ethopropazine on cytosolic Ca^{2+} rises in Calcium-6 loaded platelets (200×10^9/L), stimulated with a low dose of CRP (5 µg/mL) or thrombin (2 nM). Data are presented as effect percentages on the maximal fluorescence signal. *(d-f)*, Viability of calcein-loaded platelets (50×10^9/L) incubated with vehicle (DMSO 0.5%), ANO61, AF299 or ethopropazine (1-100 µM). Data are mean fluorescence intensities (MFI); high values indicating lack of dye leakage. Means ± SD (n = 3-4 donors), **P*<0.05, ***P*<0.01, ****P*<0.001, *****P*<0.0001 vs. vehicle, one-way ANOVA with Tukey post-hoc test.

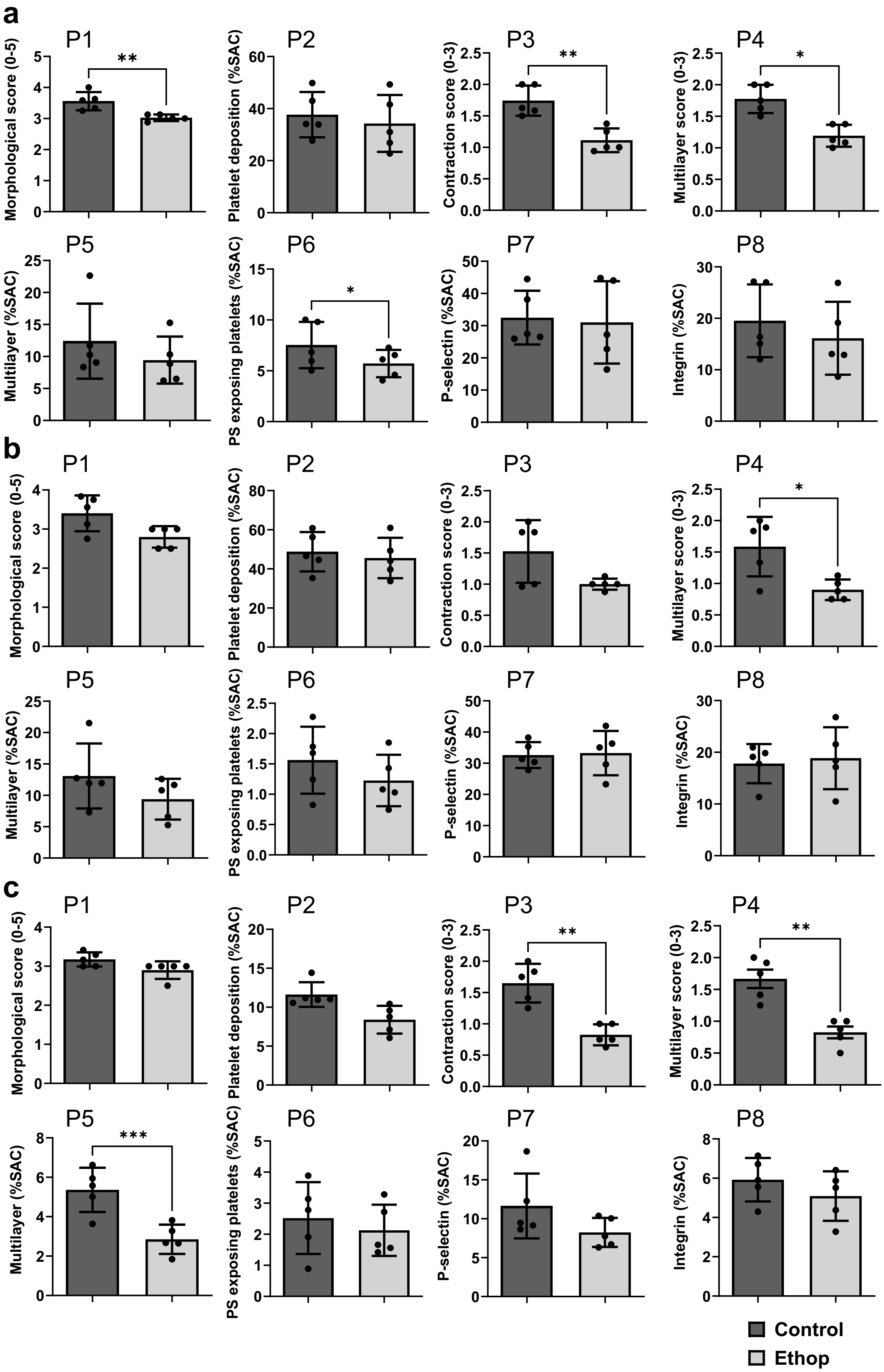
a
P1
P2
P3
P4
P5
P6
P7
P8
Morphological score (0-5)
Platelet deposition (%SAC)
Contraction score (0-3)
Multilayer score (0-3)
Multilayer (%SAC)
PS exposing platelets (%SAC)
P-selectin (%SAC)
Integrin (%SAC)
b
c
Control
Ethop

Figure S7. **Quantification of ethopropazine effect on parameters of whole-blood thrombus formation under flow.** Whole blood thrombus formation was performed, as described for Figure 6. Histograms of parameter quantifications of thrombi formed in the presence of ethopropazine (32 μM, Ethop), *vs.* control for (*a*) collagen (100 μg/mL, M1), (*b*) collagen III (100 μg/mL, M2), (*c*) and collagen III (30 μg/mL, M3) per parameter (*P1-8*). Parameters are defined as: *P1*, thrombus morphological score *P2*, platelet surface area coverage (SAC%); *P3*, thrombus contraction score; *P4*, thrombus multilayer score, and *P5*, platelet multilayer coverage (SAC%); *P6*, phosphatidylserine exposure (SAC%); *P7*, P-selectin expression (SAC%); and *P8*, integrin αIIbβ3 activation (SAC%). Data are means ± SD (n=3-5 donors), *P<0.05, **P<0.01, ***P<0.001, vs. vehicle, paired Student t-test.

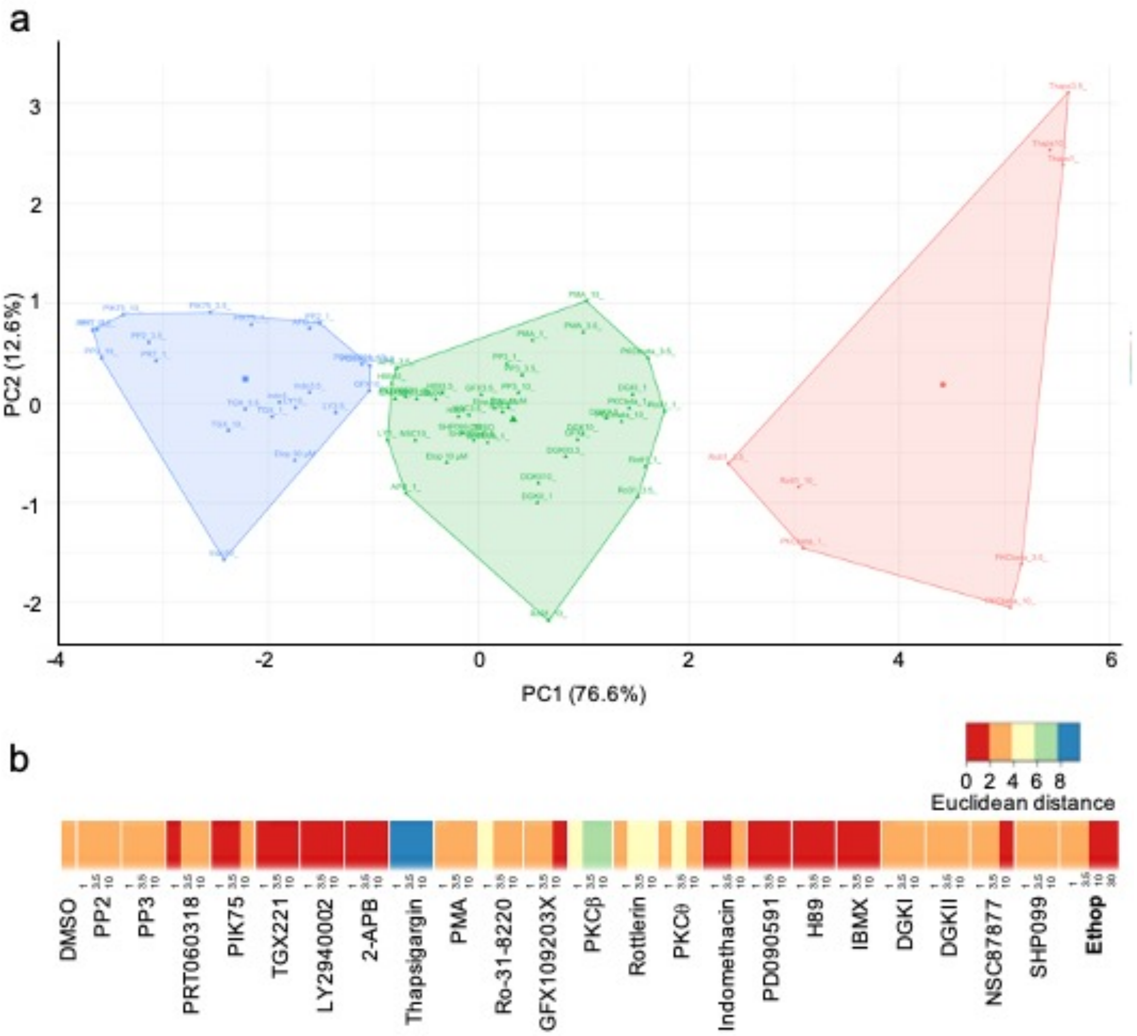

Figure S8. Prediction analysis of signaling pathways inhibited by ethopropazine. *(a),* K-means, principal component analysis (PCA) and cluster analysis combining effects of 22 reference compounds and ethopropazine (10 and 30 μM, in bold) on CRP-induced Ca^{2+} rises in platelets (curve parameters *P1-6*). *(b),* Euclidean distance matrix of k-means, indicating per reference compound high (red) or low (blue) similarity of altered $[Ca^{2+}]_i$ curves in comparison to ethopropazine effects.

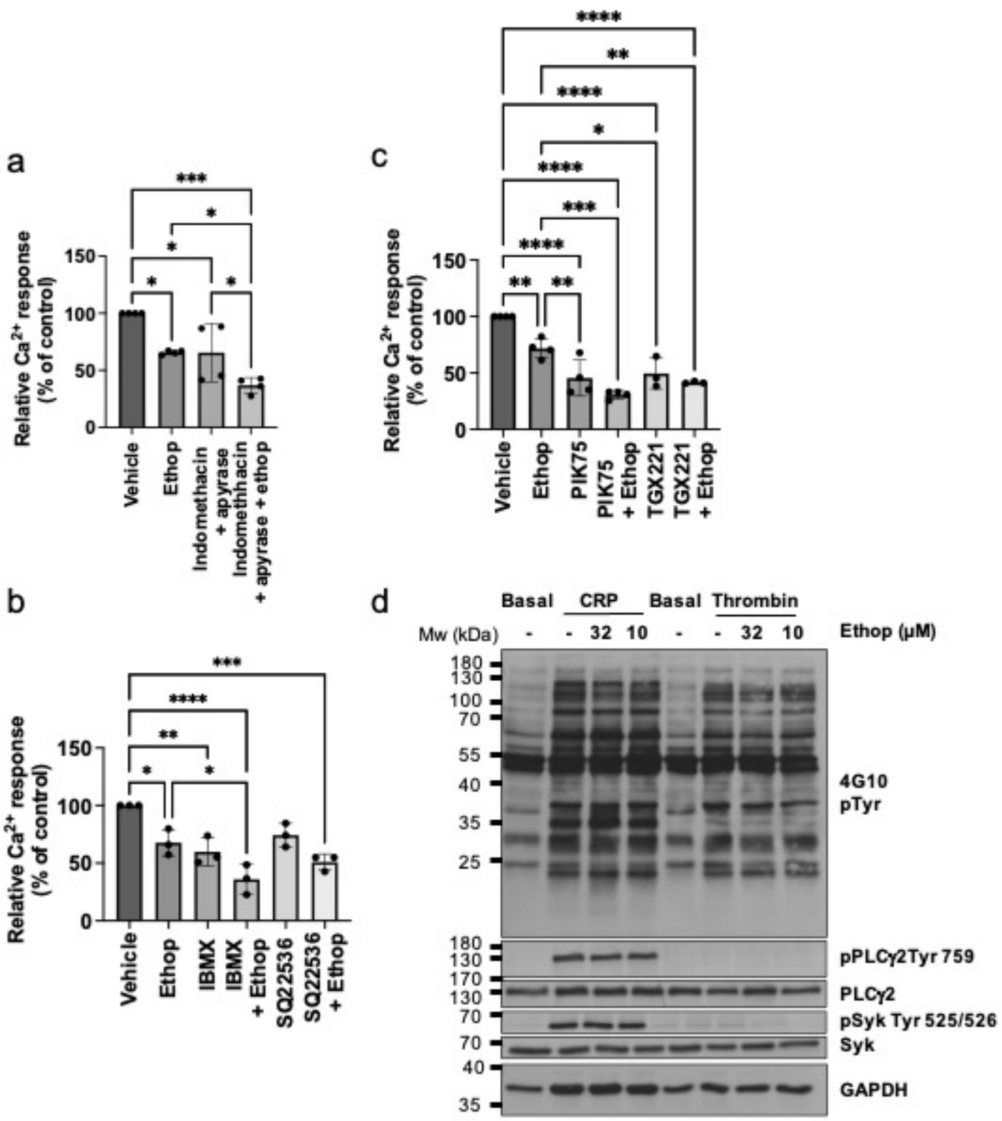

Figure S9. Ethopropazine effect on platelets independent of secondary mediators, Gi or tyrosine phosphorylation. Calcium-6 loaded platelets (200 x 10^9/L) in 96-well plates were stimulated with CRP (10 µg/mL), and Ca^{2+} responses were measured using a FlexStation 3. Pretreatment was for 10 min with vehicle medium, ethopropazine (Ethop, 10 µM), indomethacin (10 µM), apyrase (100 units/l), IBMX (20 µM), SQ22536 (100 µM), PIK75 (1 µM), TGX221 (3.5 µM) or combinations, as indicated. Fluorescence traces were quantified as rises in $[Ca^{2+}]_i$ over 10 min. *(a)* Effect of blocking secondary mediators TXA_2 and P2Y receptors by indomethacin and apyrase. *(b)* Effect on Gi pathway blocking by IBMX or by adenylyl cyclase inhibitor SQ22536. *(c)* Effect of blocking PI3K p110α with PIK75 or PI3K p110β with TGX221. *(d)* Effect of ethopropazine on protein tyrosine phosphorylation. The panel shows representative immunoblot from 4 independent experiments of platelet lysates (400 × 10^9/L), pre-treated with vehicle or ethopropazine (32 or 10 µM) and stimulated with CRP (10 µg/mL) or thrombin (4 nM). Lysates were run by SDS-PAGE, blotted onto a PDVF membrane, and incubated with anti-tyrosine antibody (4G10) or with antibodies against (p)PLCγ2, (p)Syk or GAPDH as a loading control. Original uncropped images of gels are available in Suppl. Datafile 2. Data are presented as mean ± SD (n = 3-4 donors). * P<0.05, **P<0.01, ***P<0.001, ****P<0.0001, one-way ANOVA with Tukey post-hoc test.

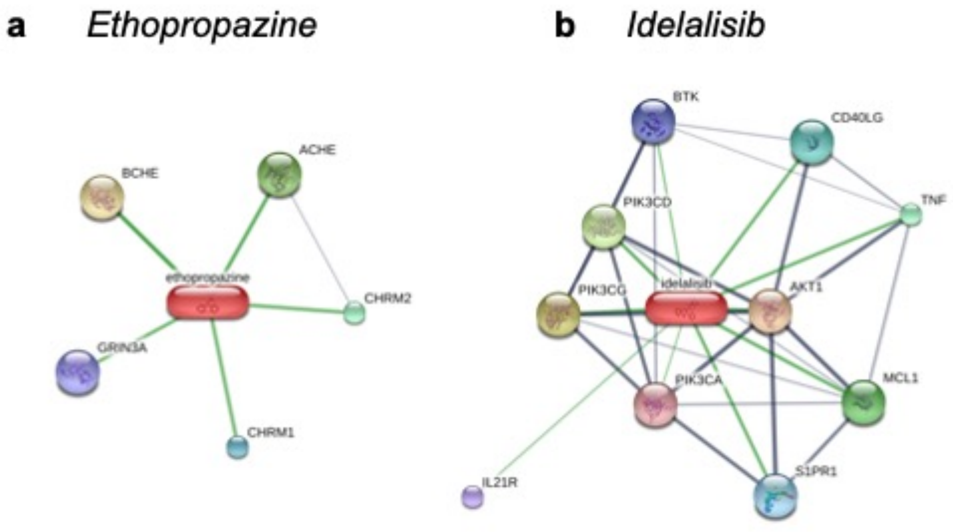

Figure S10. Network comparison of molecular targets for ethopropazine and PI3Kδ inhibitor idelalisib. Representation of String analysis exported from Stitch (http://stitch.embl.de). Colored nodes represent reported first shell interactors. *(a)* For ethopropazine, these are BCHE (butyrylcholinesterase), ACHE (acetylcholinesterase), CHRM1 and 2 (cholinergic receptor muscarinic-type 1 and 2), and GRIN3A (ionotropic glutamate receptor 3A). *(b)* For idelasilib, these are mostly phosphoinositide kinase (PIK) catalytic subunits. Edges width in green or grey reflects confidence. CHRM2 is represented by a small node due to unknown 3D structure.

Table S1. Characteristics of the reference inhibitor panel. Indicated are known targets of the 22 reference platelet inhibitors, links to UniProtKD, and reported effects on CRP- or thrombin-induced $[Ca^{2+}]_i$ traces in platelets. For further characterization of the effects and statistical analysis, see Suppl. Datafile 1.

Inhibitor	Protein targets in platelets	UniProtKD	Effect CRP	Effect thrombin	Ref.
**PP2**	Src family kinase	Src: P12931	Yes	No	[5,6]
**PP3**	Control compound for PP2	N/A	No	No	[5,6]
**PRT-060318**	Syk kinase	Syk: P43405	Yes	No	[7,8]
**PIK-75**	PI3K p110α	α: P42336	Yes	Yes	[9]
**TGX-221**	PI3K p110β	β: P42338	Yes	No	[9,10]
**LY2940002**	pan-PI3K (α and β)	α: P42336, β: P42338	Yes	No	[9]
**2-APB**	$InsP_3$ receptor	type 1: Q14571, type 2: Q14643, type 3: Q14573	Yes	Yes	[11,12]
**Thapsigargin**	SERCA2B, SERCA3	2B: P16615, 3: Q93084	Yes	Yes	[13,14]
**PMA**	pan PKC activator	α: P17252, β: P05771, θ: Q04759, δ: Q05655	Yes	Yes	[15]
**Ro-318220 (bisindolylmaleimide IX)**	pan PKC	α: P17252, β: P05771 θ: Q04759, δ: Q05655	Yes	Yes	[16,17]
**GF-109203X**	PKCα/β1	α: P17252, β: P05771, θ: Q04759, δ: Q05655	Yes	Yes	[10,18]
**PKCβ inhibitor**	PKCβ	β: P05771	Yes	Yes	[1]
**Rottlerin**	PKCδ	δ: Q05655	No	Yes	[1]
**PKCθ inhibitor**	PKCθ	θ: Q04759	No	No	[1]
**DAG kinase inhibitor I (R59-022)**	DAG kinase and serotonin S2	α (type I): P23743, ε (type III): P52429, θ (type V): P52824	Yes	No	[19]
**DAG kinase inhibitor II (R59-949)**	DAG kinase α	α (type I): P23743, γ (type I): P49619, δ	No	No	[19]

		(type II): Q16760, κ (type II): Q5KSL6			
**Indomethacin**	COX1	1: P23219	Yes	No	[20,21]
**PD-98059**	MEK1/2	1: Q13233, 2: Q9Y2U5	Yes	No	[22,23]
**H89**	Protein kinase A (PKA)	PKA: P17612	Yes	Yes	[17]
**IBMX**	Phosphodiesterase (PDE) 3A, 3B and 5	3A: Q14432, 3B: Q13370, 5: O76074	Yes	Yes	[24]
**NSC87877**	SHP1 and 2	1: P29350, 2: Q06124	No	No	[25]
**SHP099**	SHP2	SHP2: Q06124	No	No	[26]

Table S2. Reactome pathway analysis of targets of effective inhibitors of CRP- and thrombin-induced Ca^{2+} rises in platelets. Indicated are per agonist, the Reactome pathway identifiers; pathway names; numbers and ratios of entities identified; *P*-values; false discovery rates (FDR), thrombin (Thr).

Pathway identifier	Pathway name	CRP	CRP	CRP	CRP	CRP	Thr	Thr	Thr	Thr	Thr
		#Entities found	#Entities total	Entities ratio	Entities p value	Entities FDR	#Entities found	#Entities total	Entities ratio	Entities p value	Entities FDR
**R-HSA-109582**	**Hemostasis**	19	726	0.0625	1.11E-16	2.32E-14	12	726	0.0625	1.34E-12	1.02E-10
**R-HSA-76002**	**Platelet activation, signaling and aggregation**	15	265	0.0228	1.11E-16	2.32E-14	8	265	0.0228	4.07E-10	8.13E-09
**R-HSA-114508**	**Effects of PIP2 hydrolysis**	8	27	0.0023	1.22E-15	1.27E-13	5	27	0.0023	1.99E-10	4.58E-09
**R-HSA-4420097**	**VEGFA-VEGFR2 pathway**	10	100	0.0086	1.04E-14	8.66E-13	8	100	0.0086	1.83E-13	2.80E-11
**R-HSA-194138**	**Signaling by VEGF**	10	110	0.0095	2.66E-14	1.68E-12	8	110	0.0095	3.90E-13	3.98E-11
**R-HSA-5218921**	**VEGFR2 mediated cell proliferation**	7	20	0.0017	2.85E-14	1.68E-12	6	20	0.0017	1.28E-13	2.80E-11
**R-HSA-388396**	**GPCR downstream signaling**	15	637	0.0548	5.22E-13	2.71E-11	11	637	0.0548	1.49E-11	7.58E-10
**R-HSA-372790**	**Signaling by GPCR**	15	712	0.0612	2.58E-12	1.19E-10	11	712	0.0612	4.93E-11	1.48E-09
**R-HSA-416476**	**Gαq signaling events**	10	219	0.0188	2.27E-11	9.30E-10	7	219	0.0188	4.74E-09	7.12E-08
**R-HSA-162582**	**Signal transduction**	22	2592	0.2230	2.63E-11	9.99E-10	15	2592	0.2230	1.67E-10	4.52E-09
**R-HSA-1489509**	**DAG and InsP3 signaling**	6	41	0.0035	4.17E-10	1.42E-08	6	41	0.0035	9.37E-12	5.72E-10
**R-HSA-9006934**	**Signaling by receptor tyrosine kinases**	12	541	0.0465	5.40E-10	1.73E-08	9	541	0.0465	3.97E-09	6.34E-08
**R-HSA-9006925**	**Intracellular signaling by second messengers**	10	321	0.0276	9.12E-10	2.64E-08	7	321	0.0276	6.48E-08	8.42E-07
**R-HSA-112043**	**PLC beta mediated events**	6	49	0.0042	1.20E-09	3.24E-08	6	49	0.0042	2.72E-11	1.17E-09
**R-HSA-5578775**	**Ion homeostasis**	6	54	0.0046	2.14E-09	5.13E-08	6	54	0.0046	4.85E-11	1.48E-09
**R-HSA-112040**	**G-protein mediated events**	6	54	0.0046	2.14E-09	5.13E-08	6	54	0.0046	4.85E-11	1.48E-09
**R-HSA-2029480**	**Fcgamma receptor dependent phagocytosis**	8	175	0.0151	3.23E-09	7.44E-08	5	175	0.0151	2.05E-06	1.84E-05
**R-HSA-2029485**	**Role of phospholipids in phagocytosis**	7	114	0.0098	4.87E-09	1.02E-07	5	114	0.0098	2.51E-07	2.76E-06
**R-HSA-418360**	**Platelet calcium homeostasis**	5	28	0.0024	5.11E-09	1.02E-07	5	28	0.0024	2.39E-10	5.01E-09
**R-HSA-2454202**	**Fc epsilon receptor signaling**	8	218	0.0188	1.77E-08	3.36E-07	5	218	0.0188	5.95E-06	4.76E-05
**R-HSA-8853659**	**RET signaling**	5	41	0.0035	3.37E-08	6.41E-07					
**R-HSA-418346**	**Platelet homeostasis**	6	88	0.0076	3.80E-08	6.85E-07	6	88	0.0076	8.88E-10	1.69E-08

**R-HSA-111885**	**Opioid signaling**	6	90	0.0077	4.34E-08	7.38E-07	6	90	0.0077	1.02E-09	1.83E-08
**R-HSA-5607764**	**CLEC7A (dectin-1) signaling**	6	98	0.0084	7.15E-08	1.14E-06	4	98	0.0084	6.40E-06	5.12E-05
**R-HSA-5621481**	**C-type lectin receptors**	7	174	0.0150	8.62E-08	1.38E-06	5	174	0.0150	1.99E-06	1.79E-05
**R-HSA-397014**	**Muscle contraction**	7	204	0.0175	2.51E-07	3.77E-06	7	204	0.0175	2.91E-09	4.95E-08
**R-HSA-9664323**	**FCGR3A-mediated IL10 synthesis**	6	128	0.0110	3.40E-07	4.75E-06	4	128	0.0110	1.82E-05	1.16E-04
**R-HSA-5576891**	**Cardiac conduction**	6	130	0.0112	3.72E-07	5.20E-06	6	130	0.0112	8.98E-09	1.26E-07
**R-HSA-422356**	**Regulation of insulin secretion**	5	79	0.0068	8.46E-07	1.10E-05	5	79	0.0068	4.11E-08	5.35E-07
**R-HSA-9664433**	**Leishmania parasite growth and survival**	6	168	0.0145	1.64E-06	2.13E-05	4	168	0.0145	5.24E-05	3.14E-04
**R-HSA-9662851**	**Anti-inflammatory response favoring Leishmania parasite infection**	6	168	0.0145	1.64E-06	2.13E-05	4	168	0.0145	5.24E-05	3.14E-04
**R-HSA-381676**	**Glucagon-like peptide-1 regulates insulin secretion**	4	43	0.0037	2.62E-06	3.15E-05	4	43	0.0037	2.47E-07	2.76E-06
**R-HSA-512988**	**Interleukin-3, interleukin-5 and GM-CSF signaling**	4	48	0.0041	4.04E-06	4.44E-05					
**R-HSA-163685**	**Integration of energy metabolism**	5	109	0.0094	4.04E-06	4.45E-05	5	109	0.0094	2.01E-07	2.41E-06
**R-HSA-418597**	**Gαz signaling events**	4	49	0.0042	4.38E-06	4.82E-05	4	49	0.0042	4.15E-07	4.15E-06
**R-HSA-418594**	**Gαi signaling events**	7	317	0.0273	4.67E-06	5.14E-05	6	317	0.0273	1.66E-06	1.50E-05
**R-HSA-1227986**	**Signaling by ERBB2**	4	56	0.0048	7.40E-06	7.40E-05					
**R-HSA-4086398**	**Ca^{2+} pathway**	4	60	0.0052	9.69E-06	9.69E-05	4	60	0.0052	9.26E-07	9.26E-06
**R-HSA-3858494**	**Beta-catenin independent WNT signaling**	5	144	0.0124	1.54E-05	1.39E-04	5	144	0.0124	7.90E-07	7.90E-06
**R-HSA-9658195**	**Leishmania infection**	6	254	0.0218	1.72E-05	1.55E-04	4	254	0.0218	2.56E-04	1.03E-03
**R-HSA-1280218**	**Adaptive immune system**	10	958	0.0824	2.22E-05	2.00E-04	7	958	0.0824	9.15E-05	4.58E-04
**R-HSA-168249**	**Innate immune system**	11	1201	0.1033	2.51E-05	2.01E-04	7	1201	0.1033	3.81E-04	1.52E-03
**R-HSA-983705**	**Signaling by the B cell receptor**	5	176	0.0151	4.01E-05	3.21E-04	4	176	0.0151	6.27E-05	3.14E-04
**R-HSA-983695**	**Antigen activates B cell receptor leading to generation of second messengers**	4	95	0.0082	5.77E-05	4.04E-04					
**R-HSA-112314**	**Neurotransmitter receptors and postsynaptic signal transmission**	5	208	0.0179	8.81E-05	5.29E-04	4	208	0.0179	1.19E-04	5.97E-04
**R-HSA-2871809**	**FCERI mediated Ca^{2+} mobilization**	4	117	0.0101	1.28E-04	7.71E-04					
**R-HSA-6811558**	**PI5P, PP2A and IER3 regulate PI3K/AKT signaling**	4	118	0.0102	1.33E-04	7.96E-04					
**R-HSA-199418**	**Negative regulation of the PI3K/AKT network**	4	125	0.0108	1.65E-04	8.27E-04					
**R-HSA-422475**	**Axon guidance**	7	558	0.0480	1.72E-04	8.59E-04					
**R-HSA-9675108**	**Nervous system development**	7	584	0.0502	2.27E-04	0.0011					

**R-HSA-166520**	**Signaling by NTRKs**	4	139	0.0120	2.48E-04	0.0012					
**R-HSA-112315**	**Transmission across chemical synapses**	5	273	0.0235	3.11E-04	0.0013	4	273	0.0235	3.37E-04	1.35E-03
**R-HSA-418555**	**Gαs signaling events**	4	161	0.0138	4.31E-04	0.0017					
**R-HSA-195721**	**Signaling by WNT**	5	299	0.0257	4.71E-04	0.0019	5	299	0.0257	2.72E-05	1.63E-04
**R-HSA-194315**	**Signaling by Rho GTPases**	7	678	0.0583	5.63E-04	0.0023	5	678	0.0583	1.24E-03	4.94E-03
**R-HSA-9716542**	**Signaling by Rho GTPases, miro GTPases and RhoBTB3**	7	694	0.0597	6.47E-04	0.0025	5	694	0.0597	1.37E-03	5.02E-03
**R-HSA-5683057**	**MAPK family signaling cascades**	5	338	0.0291	8.20E-04	0.0025					
**R-HSA-168256**	**Immune system**	12	2261	0.1945	0.0018	0.0055					
**R-HSA-112316**	**Neuronal system**	5	419	0.0360	0.0021	0.0064	4	419	0.0360	1.67E-03	5.02E-03
**R-HSA-1257604**	**PIP3 activates AKT signaling**	4	281	0.0242	0.0033	0.0084					
**R-HSA-5673001**	**RAF/MAP kinase cascade**	4	292	0.0251	0.0038	0.0084					
**R-HSA-195258**	**Rho GTPase effectors**	4	295	0.0254	0.0040	0.0084					
**R-HSA-5684996**	**MAPK1/MAPK3 signaling**	4	299	0.0257	0.0042	0.0084					
