# Supplementary material for: Ultra-high throughput-based screening for the discovery of antiplatelet drugs affecting receptor dependent calcium signaling dynamics
Source: Sci Rep. 2024 Mar 14;14:6229. doi: 10.1038/s41598-024-56799-4 (PMC10940705; doi:10.1038/s41598-024-56799-4)
Supplement: Supplementary file 3 — Supplementary Figure S9. [file 41598_2024_56799_MOESM3_ESM.pdf]

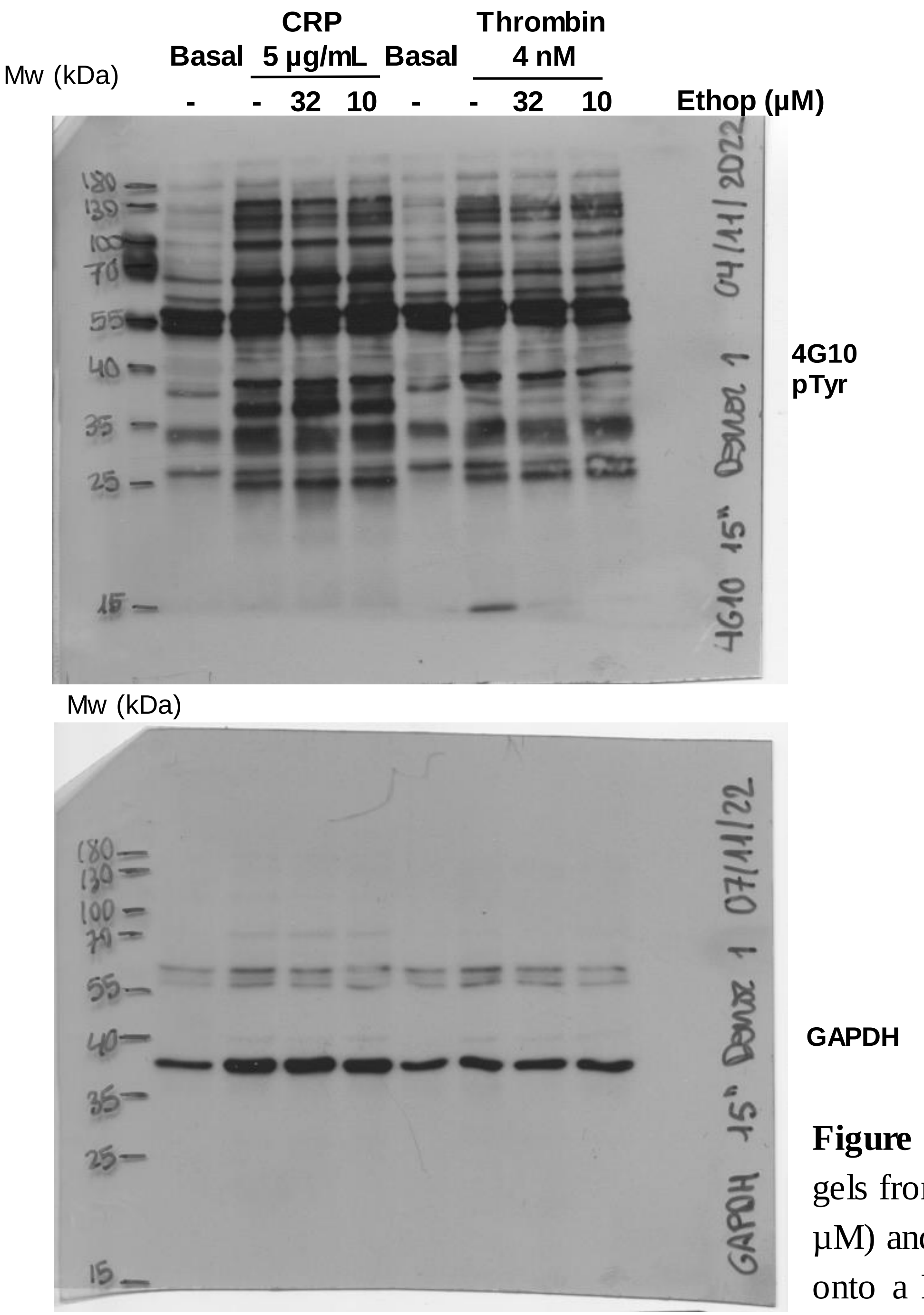

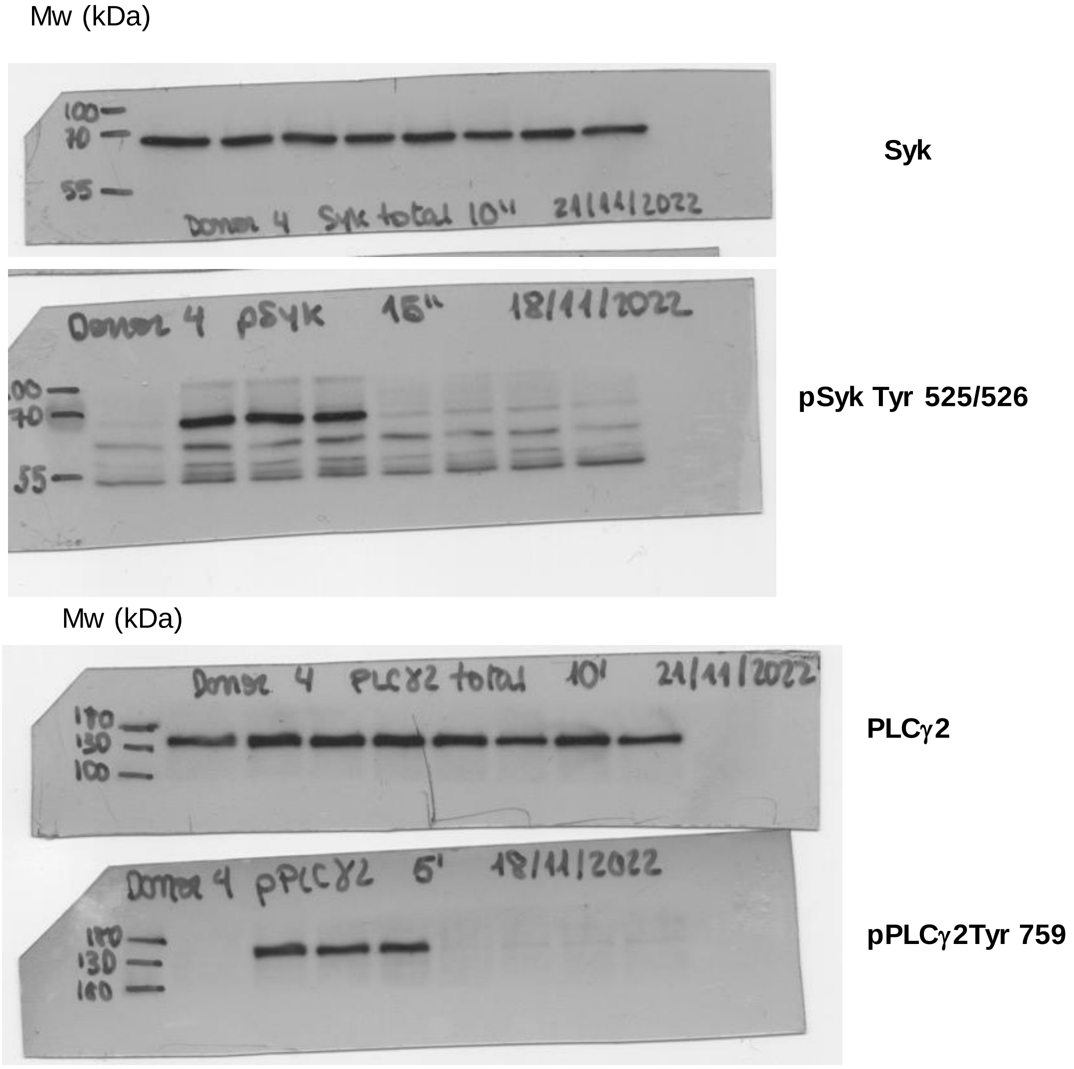

Figure S9d Effect of ethopropazine on protein tyrosine phosphorylation. Original uncropped images of gels from Figure S9d of platelet lysates (400 x 10^9/L), pre-treated with vehicle or ethopropazine (32 or 10 µM) and stimulated with CRP (10 µg/mL) or thrombin (4 nM). Lysates were run by SDS-PAGE, blotted onto a PDVF membrane, and incubated with anti-tyrosine antibody (4G10) or with antibodies against (p)PLCγ2, (p)Syk or GAPDH as a loading control.
